# Water Interaction with Fe_2_NiP Schreibersite (110) Surface: a Quantum Mechanical Atomistic Perspective

**DOI:** 10.1021/acs.jpcc.1c09947

**Published:** 2022-01-25

**Authors:** Stefano Pantaleone, Marta Corno, Albert Rimola, Nadia Balucani, Piero Ugliengo

**Affiliations:** † Dipartimento di Chimica and Nanostructured Interfaces and Surfaces (NIS) Centre, Università degli Studi di Torino, via P. Giuria 7, I-10125, Torino, Italy; ‡ Dipartimento di Chimica, Biologia e Biotecnologie, Università degli Studi di Perugia, Via Elce di Sotto 8, I-06123 Perugia, Italy; § Departament de Química, Universitat Autònoma de Barcelona, 08193 Bellaterra, Catalonia Spain; ∥ Osservatorio Astrofisico di Arcetri, Largo E. Fermi 5, I-50125 Firenze, Italy; ⊥ Université Grenoble Alpes, CNRS, Institut de Planétologie et d’Astrophysique de Grenoble (IPAG), F-38000 Grenoble, France

## Abstract

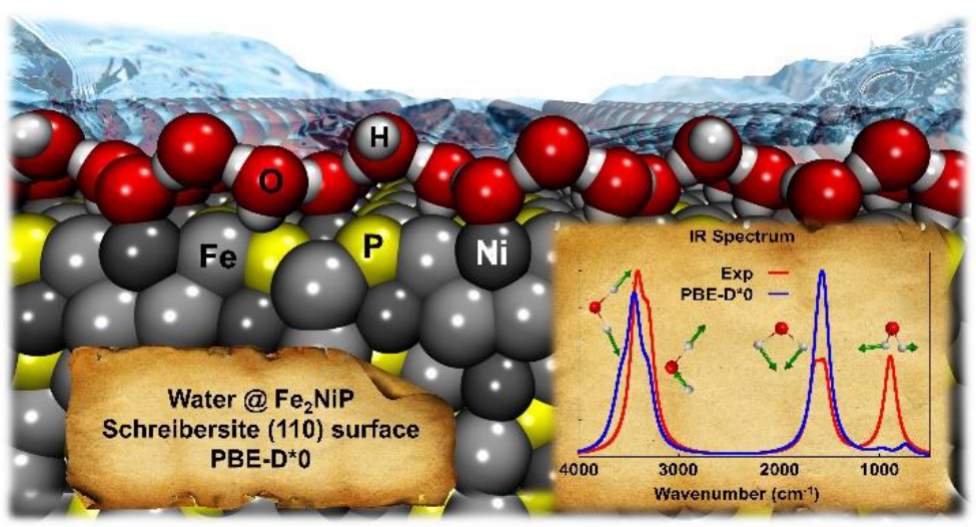

Phosphorus is an element of primary importance for all living creatures, being present in many biological activities in the form of phosphate (PO_4_^3–^). However, there are still open questions about the origin of this specific element and on the transformation that allowed it to be incorporated in biological systems. The most probable source of prebiotic phosphorus is the intense meteoritic bombardment during the Archean era, a few million years after the solar system formation, which brought tons of iron-phosphide materials (schreibersite) on the early Earth crust. It was recently demonstrated that by simple wetting/corrosion processes from this material, various oxygenated phosphorus compounds are produced. In the present work, the wetting process of schreibersite (Fe_2_NiP) was studied by computer simulations using density functional theory, with the PBE functional supplemented with dispersive interactions through a posteriori empirical correction. To start disentangling the complexity of the system, only the most stable (110) surface of Fe_2_NiP was used simulating different water coverages, from which structures, water binding energies, and vibrational spectra have been predicted. The computed (ana-)harmonic infrared spectra have been compared with the experimental ones, thus, confirming the validity of the adopted methodology and models.

## 1 Introduction

Naturally occurring iron–nickel phosphides are in the mineral form of schreibersite (Fe,Ni)_3_P, which is present as a minor phase of iron meteorites,^1−3^ whose heavy elements are the first that condensate from solar nebulae.^4^

Phosphorus is present in living systems in the form of phosphate (PO_4_^3–^) and, despite its low abundance (1%) with respect to the other macro-elements (SONCH), it is ubiquitous, in nucleic acids (DNA and RNA), in molecules that provide energy for metabolic processes (ATP, ADP, AMP), and in phospholipids, among others. The most abundant source of phosphorus on Earth is the mineral apatite (Ca_5_(PO_4_)_3_(F,OH,Cl), which, however, has extremely low solubility and reactivity, and therefore, it can hardly support the presence of the phosphate groups in living organisms.^5^ As a consequence, other possible sources of reduced and reactive phosphorus were investigated in the last decades.

In 1955, Gulick postulated the release of phosphates by corrosion reactions operated by water contact with the surface of schreibersite.^6^ Indeed, during the Archean era (4.0–3.8 billion years ago), just after the solar system formation, a large quantity of small bodies (meteorites, comets, and other asteroidal bodies) hit the planet, thus, brining all the organic matter encapsulated inside.^7−9^

In 2005, Pasek et al. carried out the first water corrosion experiment on a model of schreibersite, Fe_3_P, in which they demonstrated the production of different phosphorus oxygenated compounds (phosphates, phosphites, hypophosphites), and also their reaction with organic molecules.^10^ Recent models estimate that about 1–10% of the early Earth crust was composed by phosphide minerals and that a meteorite of about 60 tons should produce about 1 ton of reduced phosphorus.^11^ Another route recently proposed by Hess et al. implies the in situ formation of schreibersite by light-activated processes in clay-rich soils.^12^ According to this study, 10–1000 kg/year of phosphide and 100–10000 kg/year of phosphite and hypophosphite could be produced, ensuring a continuous availability of reduced phosphorus without depending on the meteor flux. Therefore, the reactivity of schreibersite toward water is a key step for biological phosphorus incorporation.

The first (Fe,Ni)_3_P water corrosion experiment by Pasek et al. encouraged many subsequent works;^13−19^ in some cases, organic species were added in the experiment,^16^ showing the tendency of reduced phosphorus to activate phosphorylation reactions, also producing nucleosides^14^ and complex sugars.^19^ Experimentally, the wetting process of schreibersite was investigated by means of RAIRS (Reflection–Absorption InfraRed Spectroscopy) at both different temperatures (120–298 K) and water coverages (from 1 to 100 Langmuir, 1 Langmuir representing the monolayer).^13,17^ These studies showed that the H_2_O molecules preferentially bind to the P atoms at the schreibersite surface through a direct P···O interaction, also confirmed by addition of isotopic water-^18^O.^18^ Nevertheless, an atomistic perspective of the whole water corrosion process of schreibersite (both from the point of view of the adsorption and the reactivity) is still elusive, as, to our knowledge, no computational work has been performed on this specific topic. Some studies were carried out on the “father” of schreibersite, Fe_3_P, studying the phase stability of different polymorphs in order to understand if it is present, and in which form, in planetary cores.^20−22^

A recent computational work on the bulk and surface structures and properties of schreibersite was published by us.^23^ According to that, it is unlikely that the oxygen of H_2_O (partially negatively charged) interacts with the surface P atoms, which also bring a partial (close to −1e) negative charge,^23−26^ thus, resulting in an electrostatic repulsion. In the present paper, we simulate the adsorption of water on the most stable facet of schreibersite, namely, the {110} exploring different water’s coverage regimes, up to a water multilayer. Moreover, also the water deprotonation was considered to explore the very first step of the route toward the formation of phosphorus oxygenated species. The most reactive surfaces will be the object of future work.

## 2 Computational Details

The adsorption of water on schreibersite was studied by means of periodic DFT calculations carried out with the Vienna Ab-initio Simulation Package (VASP) code,^27−30^ which uses projector-augmented wave (PAW) pseudopotentials^31^ to describe the ionic cores and a plane wave basis set for the valence electrons. The same approach was used to characterize the pristine schreibersite.^23^

Geometry optimizations and frequency calculations were performed with the gradient corrected PBE functional,^32^ with a posteriori Grimme D2 correction,^33^ modified for solids (D*).^34^ Moreover, C6 atomic coefficients related to polarizabilities on Fe and Ni metal atoms were set to 0 (i.e., no dispersion interaction contribution from metal atoms). This setup was chosen according to the best results obtained in our previous work on the bulk and bare surfaces of schreibersite.^23^ On O and H atoms, the original D* parameters were used. This method of choice is referred to as PBE-D*0 along the work. The cutoff energy of plane waves (which control the accuracy of the calculations) was set to 500 eV. The self-consistent field (SCF) iterative procedure was converged to a tolerance in total energy of Δ*E* = 10^–5^ eV for geometry optimizations, while for frequency calculations, the tolerance was decreased to Δ*E* = 10^–6^ eV. The tolerance on gradients during the optimization procedure was set to 0.01 eV/Å for each atom in each direction. The Monkhorst–Pack sampling of the Brillouin zone was used for the *k*-points mesh. Shrinking factors for the (110) surface have been set on (8 8 1) on the unit cell (*a* = 4.374 Å, *b* = 6.719 Å, γ = 108.998°), while they were reduced to (4 8 1) and (4 4 1) for 2 × 1 and 2 × 2 supercell models (for a total number of 64 and 16 *k*-points, respectively). The shrinking factor related to the nonperiodic direction was always set to “1” (i.e., no sampling of the reciprocal space). As VASP relies on the plane waves basis set and, accordingly, surfaces are replicated also along the nonperiodic direction, the vacuum space among fictitious replicas was set to at least 20 Å to minimize the interactions among replica images. Therefore, the final *c* cell axis was set to 40 Å. The relaxation of water adsorbed at the (110) surface was carried out by moving all atoms in the unit cell while keeping the cell parameters fixed at the geometry optimized for the bare surface to enforce the rigidity due to the underneath bulk.

Adsorption energies (AE) of water adsorbed on the (110) shreibersite surface (water was only adsorbed on one of the two equivalent (110) faces of schreibersite) were calculated as
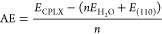
where *E*_CPLX_ is the energy of the complex (water adsorbed on the surface), *E*_H_2_O_ and *E*_(110)_ are the energies of the isolated water molecule and the (110) bare surface, each one calculated at its relaxed geometry, while *n* is the number of adsorbed water molecules per surface unit cell. The corresponding adsorption enthalpy (AH) and free energy (AG) were calculated by adding the proper thermodynamic corrections. When considering water dissociation at the (110) surface, the reaction energy is computed with respect to the most stable molecular adsorption and labeled DE, as well as the corresponding thermic corrections to the energy (DH and DG). Please note that negative values of AE and DE mean thermodynamic favorable processes.

Vibrational frequencies were computed at the Γ point by numerical differentiation of the analytical first derivatives using the central difference formula (i.e., two displacements of 0.02 Å for each atom in each *x*-, *y*-, and *z*-direction), in order to confirm that the optimized structure is a minimum (all real frequencies) and to apply thermal corrections to the calculated energies. To simulate IR spectra, the Phonopy^35^ code was used for both generating atomic displacements and processing VASP outputs. To plot IR spectra, the convolution of intensities was done with Lorentzian functions and a fwhm (full width at half maximum) of 50 cm^–1^. Thermochemistry has been corrected using the quasi-harmonic approximation, proposed by Grimme,^36^ in which frequencies lower than the 100 cm^–1^ are replaced by free rotor modes. This improves the calculation of the thermal corrections, which would be otherwise underestimated when considering very low frequency values. To avoid discontinuity close to the cutoff, a damping function was used to interpolate the values computed within the two ranges of frequencies. To recover the systematic error due to the methodology and to the anharmonic nature of the O–H vibration, simulated O–H harmonic stretching frequencies were scaled for a proper factor calculated as
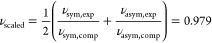
where *ν*_sym__,exp_ and *ν*_asym__,exp_ are the experimental symmetric and antisymmetric stretching of the isolated water molecule as taken from NIST, while *ν*_sym__,comp_ and *ν*_asym__,comp_ are the calculated ones in the harmonic approximation with the present methodology, on a geometry optimized single water molecule in a unit cell of *a* = 20 Å, *b* = 20 Å, and *c* = 20 Å, to ensure negligible lateral interactions among water replicas. The final scaled water monomer stretching frequencies are 3761 and 3652 cm^–1^, for the symmetric/antisymmetric modes. The bending mode at 1585 cm^–1^ was obviously unscaled. For the prediction of the infrared spectra for the considered adsorption cases, we ensure that the scaling was only applied to vibrational modes involving the OH stretching only.

Visualization and manipulation of the structures and figures rendering have been done with the MOLDRAW,^37^ VMD,^38^ and POVRAY^39^ programs.

## 3 Results

### 3.1 Molecular Water Adsorption

To study the weathering process of schreibersite, the interaction of water was only modeled on the (110) most stable schreibersite surface, as characterized in our recent work.^23^ The adsorption of water was simulated at different surface coverage regimes, from a single water molecule to the monolayer up to a thin liquid water multilayer.

Bader charge analysis in Table 1 reveals that, among the outermost atoms of the (110) schreibersite surface, Fe is the only one with a weakly positive charge (0.327 |e|), Ni preserves its atomic nature (the charge being almost zero), while P brings a negative charge (−0.577 |e|). In view of these results, the adsorption of a water molecule is expected to take place through the electrostatic complementarity principle, that is, its O (−1.178 |e|) atom will likely be adsorbed on the surface of Fe atoms through its lone pairs (electrostatic plus charge transfer), while the H atoms (0.584 |e|) will interact with P atoms through a weak H-bond.

**Table 1:** Bader Charge Analysis of All the Studied Systems^a^

molecular water				deprotonated water		
atom	net charge	charge diff	net transfer	atom	net charge	charge diff
**(110)**				**Fe/NiH-FeOH**		
P	–0.577			H–Fe_Ni	–0.240	–0.824
Fe	0.327			H–OH	0.579	–0.005
Ni	–0.043			O	–1.023	0.145
**H_2_O gas**				Fe29–H	0.420	0.093
O	–1.168			Ni76–H	0.065	0.108
H	0.584			Fe28–OH	0.677	0.350
**H_2_O–Ni 1×1**				**FeH-Fe/Ni–OH**		
H	0.595	0.011	0.017	H–Fe	–0.167	–0.750
H	0.629	0.045		H–OH	0.565	–0.019
O	–1.206	–0.038		O	–1.073	0.095
Ni	0.125	0.169		Fe28–H	0.285	–0.042
P10	–0.651	–0.074		Fe29–OH	0.591	0.265
P11	–0.638	–0.061		Ni76–OH	0.275	0.319
**H_2_O–Ni 2×1**				**Fe/NiH-POH**		
H	0.616	0.032	0.033	H–OH	0.593	0.009
H	0.613	0.029		H–Fe_Ni	–0.257	–0.841
O	–1.196	–0.028		O	–1.302	–0.134
Ni	0.170	0.213		P–OH	0.301	0.878
P10	–0.641	–0.064		Fe41–H	0.428	0.101
P11	–0.607	–0.030		Ni82–H	–0.035	0.009
**H**_**2**_**O–Ni 2×2**				**Fe/NiH-POH_2**		
H	0.604	0.020	0.040	H–Fe	–0.249	–0.833
H	0.611	0.027		H–OH	0.617	0.033
O	–1.175	–0.007		O	–1.298	–0.130
Ni	0.174	0.217		P–OH	0.233	0.810
P10	–0.639	–0.061		Fe29–H	0.385	0.059
P11	–0.616	–0.039		Ni76–H	0.039	0.083
**H**_**2**_**O–Fe 1×1**				**Fe/NiH-POH_3**		
H	0.605	0.021	–0.012	H–Fe	–0.242	–0.826
H	0.606	0.022		H–OH	0.608	0.024
O	–1.223	–0.055		O	–1.291	–0.123
Fe	0.455	0.128		P–OH	0.333	0.910
P7	–0.584	–0.007		Fe53–H	0.359	0.032
P8	–0.600	–0.022		Ni89–H	0.025	0.068
**H**_**2**_**O–Fe 2×1**				**PH-Ni/FeOH**		
H	0.604	0.020	0.010	H–P	–0.325	–0.909
H	0.615	0.031		H–OH	0.568	–0.016
O	–1.209	–0.041		O	–1.085	0.083
Fe	0.467	0.140		P–H	–0.082	0.495
P4	–0.586	–0.009		Fe40-OH	0.590	0.263
P5	–0.626	–0.049		Ni82-OH	0.157	0.201
**H**_**2**_**O–Fe 2×2**				**PH-Ni/FeOH_2**		
H	0.597	0.013	0.014	H–P	–0.393	–0.977
H	0.589	0.005		H–OH	0.597	0.013
O	–1.171	–0.004		O	–1.115	0.053
Fe	0.470	0.144		P–H	–0.032	0.545
P4	–0.602	–0.025		Fe40–OH	0.565	0.238
P17	–0.620	–0.042		Ni82–OH	0.217	0.260

a Net charge, charge difference, and net transfer are in units of the electron charge and calculated as differences between charges of the actual system with respect to the corresponding bare system. In the “atom” column, the notation X–Y means that the reported charge is relative to atom X, which is bound to atom Y.

### 3.2 Single Water Adsorption

**Figure 1:**
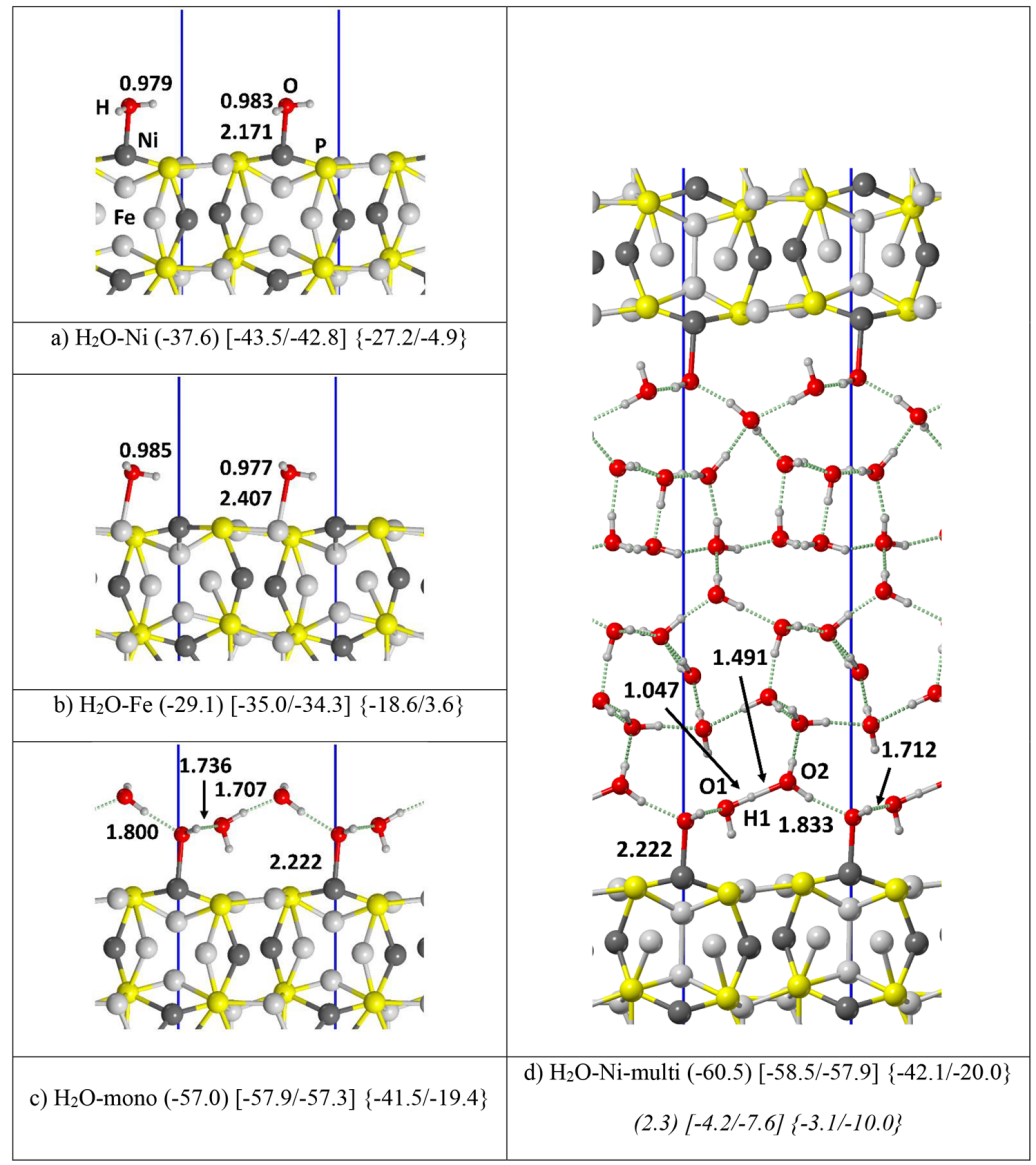
PBE-D*0 optimized structures of a single water molecule (a) and (b), water monolayer (c), and water multilayer (d), in interaction with the (110) schreibersite surface. Atoms color legend: H is in white, O is in red, P is in yellow, Fe is in light gray, and Ni is in dark gray, and the unit cell in blue. In round parentheses is the adsorption energy per water molecule in kJ/mol. In square and curly parentheses are the adsorption enthalpy and free energy at 125/298 K. Italic numbers in section (d) are computed using the water bulk as a reference. Distances are in Å.

Figure 1 reports the structures of water adsorbed on the unit cell of the schreibersite (110) slab (*a* = 4.374 Å, *b* = 6.719 Å, γ = 108.99°) and their corresponding adsorption energies. Surprisingly, the most stable adsorption mode (i.e., the one which presents the smallest adsorption energy) is for H_2_O–Ni (−37.6 kJ/mol, Figure 1a), in contrast with what was expected by adopting the naïve electrostatic complementarity principle. We ascribe this unexpected stabilization to the *d*^8^ electron configuration of the atomic Ni, which allows for the electron pair donation of the water O atom toward Ni unfilled *d* orbitals, thus, almost filling up completely the *d* manifold. This justifies the additional stabilization with respect to water adsorption on Fe (−29.1 kJ/mol), whose *d* block is still incomplete, even after water adsorption. Table 1 shows that charge transfer effects are rather subtle to rationalize. Upon adsorption, the system does not present a significant charge transfer character between the adsorbate and the surface, the difference in the atomic charges of the adsorbed water molecule being negligible with respect to the isolated one. In contrast, the charge on the Ni atom increases by 0.17 |e|. All this means that the water molecule does not exert a direct effect on the electronic structure of schreibersite; instead, its adsorption affects the coordination geometry of Ni, which ultimately produces a slight rearrangement in the global electronic structure of the surface.

### 3.3 Single Water Adsorption: Coverage Effect

**Figure 2:**
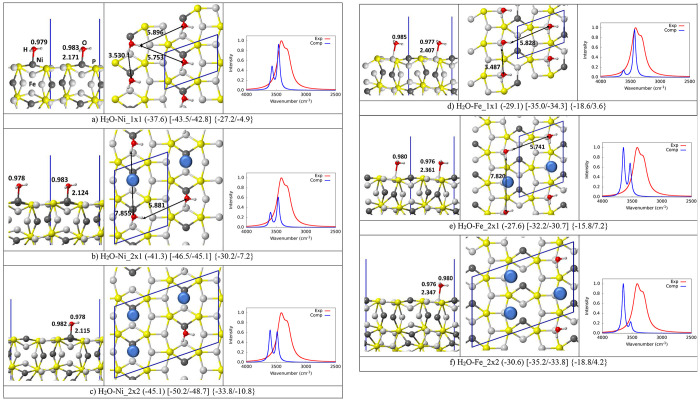
PBE-D*0 optimized structures of isolated water molecule adsorbed on different supercell sizes and corresponding IR spectra. The experimental spectrum is also shown. Adapted with permission from ref (17). Copyright 2017 ACS. Atoms color legend: H is in white, O is in red, P is in yellow, Fe is in light gray, and Ni is in dark gray, and the unit cell is in blue. The blue spheres represent the removed water molecules to simulate the decreased coverage. In round parentheses is the adsorption energy per water molecule in kJ/mol. In square and curly parentheses are the adsorption enthalpy and free energy at 125/298 K. Distances are in Å.

For a single water molecule adsorption at the (110) schreibersite surface, the lateral interaction with water adsorbed in the neighboring cells appeared to be small from the perspective of the interwater distances. Nevertheless, we checked this effect by enlarging the original adsorption cell to 2 × 1 and 2 × 2 supercells, simulating a lower water coverage. As Figure 2 shows, for both adsorptions on the Ni and Fe sites, the O–H bond distances are almost unchanged as a function of the different surface coverages, while the O–metal bond shortens with the decrease in water loading, with a corresponding decrease in the adsorption energy. This effect is less pronounced in the case of the adsorption on Fe (see Figure 2, sections d and f) than on Ni (see Figure 2, sections a–c), the O–Fe bond (2.407, 2.361, 2.347 Å, for the 1 × 1, 2 × 1, and 2 × 2 supercells, respectively) being longer than the O–Ni bond (2.165, 2.124, and 2.115 Å). The adsorption energies at the Fe site (−29.1/-27.6/–30.6 kJ/mol) are less-dependent on the water loading and more erratic compared with those computed at the Ni site (−37.6/–41.3/–45.1 kJ/mol). As already pointed out, despite the significant difference in the O–metal bond, the effect on the O–H bond length appears to be negligible.

To check more carefully on the bonding effects, we computed the vibrational frequencies for all the considered supercell models. The IR spectra of Figure 2 show that (i) the symmetric/antisymmetric OH stretching of adsorbed water suffers from a bathochromic shift by around 300 cm^–1^ with respect to the gas-phase molecule (vibrating at 3761/3652 cm^–1^); and (ii) the infrared intensities are also sensitive to the water coverage and also to the metal (please compare spectra for sections a–c with those of sections d–f). When adsorbed on Ni, the water frequency bands are less affected by the decreasing water coverage while showing a change in the relative infrared intensity. Instead, when adsorbed at Fe site, the H_2_O–Fe_1×1 case (Figure 2d) shows a definite larger bathochromic shift of both frequencies compared to both the higher water coverage and when compared to the corresponding Ni adsorption case H_2_O–Ni_1×1 (Figure 2a). Inspection of charge transfer from data in Table 1 revealed that, while for the Ni cases water always loses electronic charge toward the surface, for Fe cases the charge flux is reversed with respect to the Ni adsorption. While evaluating absolute charge transfer is dependent on the adopted methodology, the present comparison is meaningful, as it compares similar situations on closely related systems. Indeed, an increased charge transfer toward water populates the σ*(OH) orbital, hence, increasing the bathochromic shifts of the OH related stretching frequencies, in agreement with theoretical interpretation.^40^ We also see that water coverage decrease causes a hypsochromic shift of the OH vibrational bands, thus, worsening the agreement with the experiment, particularly for the Fe adsorption. This may be taken as an indication that the experimental results of ref (17) are for a water surface coverage closer to the monolayer rather than to the adsorption of well isolated water molecules.

### 3.4 Water Monolayer and Multilayer Adsorption

A water monolayer coverage was simulated by adsorbing up to three water molecules on the surface unit cell, limiting our analysis to the adsorption on the Ni site, as it is the most favorable one (Figure 1c). Results indicate that only one out of three water molecules is in direct contact with the Ni atom; the other water molecules prefer an interwater cooperative H-bond interaction, rather than being adsorbed on the Fe atoms. The cooperative H-bond interactions between water molecules, together with the contribution from the water directly adsorbed at the Ni atom, brings a more favorable adsorption energy per water molecule (−57.0 kJ/mol) compared to the single water adsorption case (−37.6 kJ/mol, vide supra).

As the last step, we simulated a water multilayer case (starting from the optimized geometry of the monolayer) by completely filling in with water molecules the vacuum space among the fictitious nonperiodic replicas of the schreibersite slab. The starting geometry of water molecules was obtained with the Packmol program.^41^ The adsorption energy per water molecule (−60.5 kJ/mol) is close to that of the monolayer (−57,0 kJ/mol), and both are similar to the reverse of the binding energy per water molecule for the pure amorphous water bulk model (AE = −62.8 kJ/mol) [AH^125/298^ = −54.4/–56.1 kJ/mol] {AG^125/298^ = −39.1/–19.5 kJ/mol}. This proves that the effect of the schreibersite surface on a water multilayer is rather moderate as their features are close to that of liquid water models. Nevertheless, when we used as a reference the amorphous water bulk instead of isolated water molecules, the adsorption process is still slightly favorable, at least for enthalpy and free energy contributions (AE = 2.3 kJ/mol) [AH^125/298^ = −4.2/–7.6 kJ/mol] {AG^125/298^ = −3.1/–10.0 kJ/mol} (see Figure 1d, italic numbers), showing a role of the surface in dictating the energetic of adsorption.

**Figure 3:**
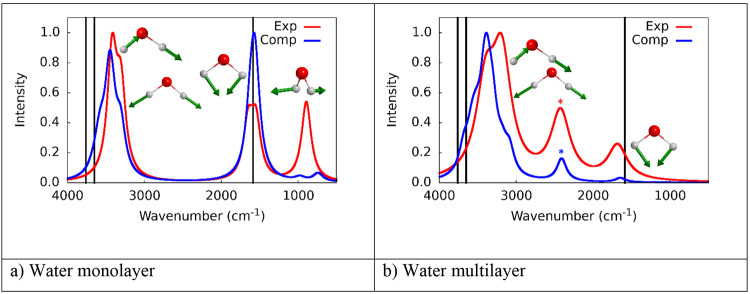
Experimental (red) and computed (blue) IR spectra. Black bars correspond to the isolated water molecule normal modes. Green arrows represent the motion of the vibrational modes. Atoms color legend: H is in white, O is in red. (a) The experimental spectrum (1.0 Langmuir, 125 K recorded T) is adapted with permission from ref (17). Copyright 2017 ACS. (b) The experimental spectrum (100 Langmuir, 295 K recorded T) is adapted with permission from ref (13). Copyright 2016 Royal Society of Chemistry.

Figure 3 shows the anharmonic (scaled, vide supra) simulated infrared spectra of both the water mono (Figure 3a) and multilayer cases (Figure 3b) in comparison with the experimental ones (taken and adapted from ref (17) for water monolayer recorded at 125 K, and from ref (13) for water multilayer recorded at 295 K). The simulated spectrum of water monolayer (Figure 3a) was built up by merging the IR spectra of single water adsorptions (H_2_O–Ni and H_2_O–Fe) in addition to the water monolayer (H_2_O–mono) to mimic the complex experimental coverage in which different water patches may populate the surface simultaneously. In contrast, the spectrum for the water multilayer case (Figure 3b) was simulated using exclusively the water multilayer contributions, as there is little ambiguity in controlling the coverage from the experiments. For the monolayer case, the agreement with the experiment is good, when considering the spectrum recorded at 125 K, despite some discrepancies in the IR relative intensities related to the rocking modes of adsorbed water. In ref (17), the authors hypothesized the formation of a water multilayer despite the maximum coverage of 1.0 Langmuir, characteristic of the water monolayer. The main difference between mono- and multilayer experimental spectra is a rather broad peak shown in Figure 3b (marked by an asterisk) at around 2423 cm^–1^, which was attributed by the authors of ref (17) to the formation of either P–H and/or P–OH bonds (i.e., to the water dissociative adsorption, see next section).^13^ At low temperatures, the predominant interaction was attributed to water molecularly adsorbed on schreibersite, through a direct interaction of the O atoms with the P atom of the surface. However, the present atomistic simulation results are in full disagreement with that model (vide supra), as we find that single water directly interacts with the metal atoms, a phenomenon that can be explained on a simple electrostatic base (vide supra). Any attempt to force the P–OH_2_ interaction by putting the water molecule close to the P atom resulted in water promptly escaping from that position during geometry optimization. The only way water will interact with the P atoms is by dissociative adsorption, in which a new P–O bond is formed (vide infra).

### 3.5 Dissociative Water Adsorption

**Figure 4:**
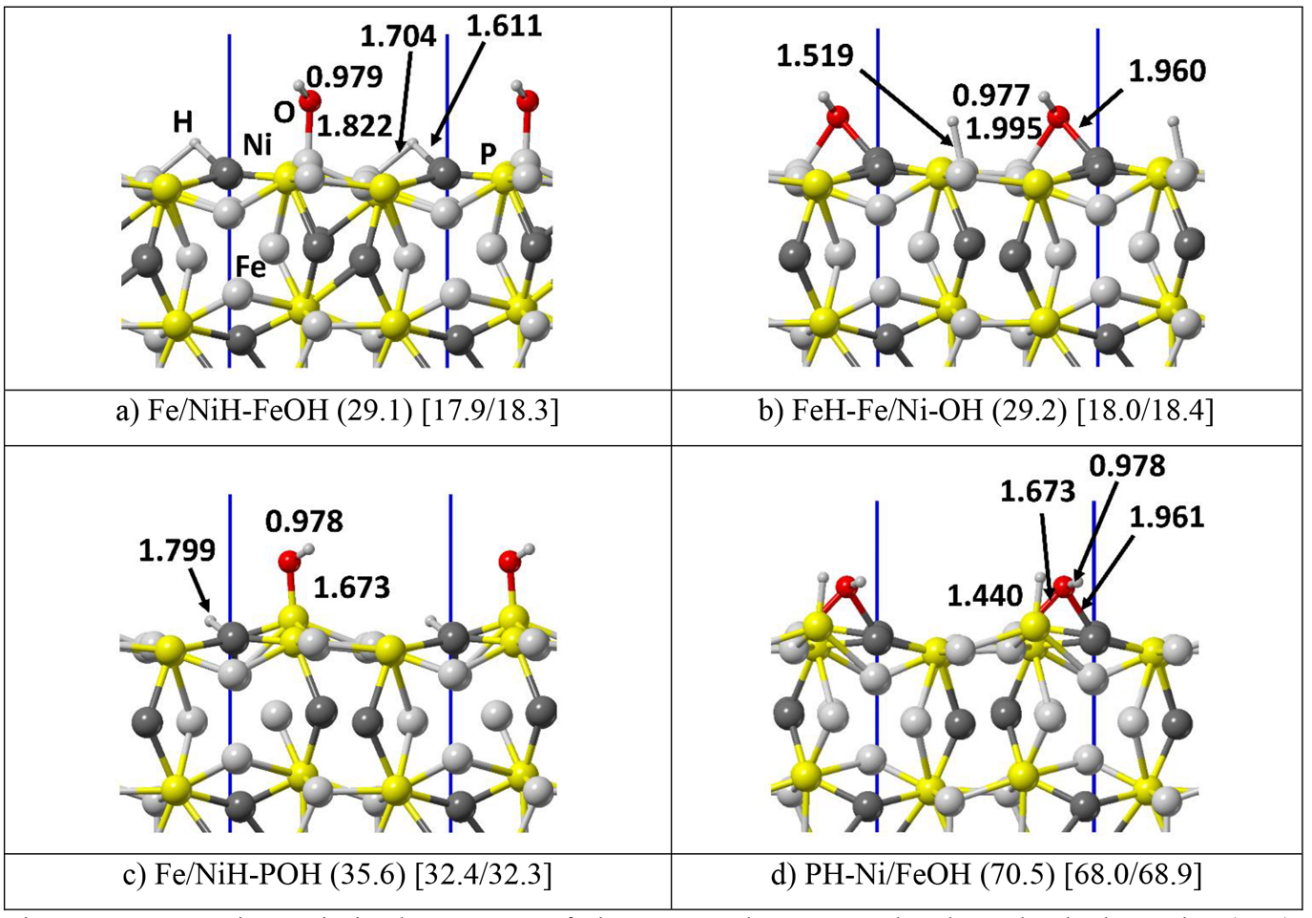
PBE-D*0 optimized structure of deprotonated water molecules adsorbed on the (110) schreibersite surface. Atoms color legend: H is in white, O is in red, P is in yellow, Fe is in light gray, and Ni is in dark gray, and the unit cell in blue. In round parentheses, the dissociation energy in kJ/mol is computed with respect to the most stable molecular adsorption case (H_2_O–Fe_2×1) assumed as a reference. In square parentheses the dissociation enthalpy at [125/298] K. Distances are in Å.

Above, only the molecular water was considered as a possible candidate toward schreibersite (110) surface adsorption. In this section, we consider the case of chemisorption, with the formation of metal–H/metal–OH and P–H/P–OH surface features by water dissociative adsorption. Figure 4 shows the possible dissociative water adsorption modes on the 2 × 1 supercell of the (110) schreibersite. Dissociative adsorption represents the very first step of the schreibersite corrosion process, where oxidized forms of each surface species are formed, the most important for biological processes being the P–O bond formation, at the cost of heterolytically breaking one OH bond of water to give OH^–^ and H^+^.

In this case, the supercell was used to increase the water fragments degrees of freedom at the (110) surface. We computed the deprotonation energies (DE) shown in Figure 4 by using, as a reference system, the energy of the most stable molecular single water adsorption (H_2_O–Ni_2×1, Figure 2b). In all cases, the water deprotonation at the surface is endothermic with respect to the molecular adsorption case, therefore requiring relative high temperatures to occur.

The following possibilities were studied here: (i) water deprotonation on metal atoms, that is, both the H and OH groups on Ni and Fe (Figure 4a,b); (ii) OH group on phosphorus and H on metal atoms (Figure 4c); and (iii) OH group on metal atoms and H on phosphorus (Figure 4d; more cases in the SI). The stability ranking is clear: the most stable situation happens when both metal atoms are saturated (i.e., both H and OH on Fe and Ni, and vice versa, Figure 4a,b) with almost indistinguishable DE values, followed by the hydroxylation of phosphorus (OH on P, H on Fe or Ni, Figure 4c), and the least stable cases are the hydriding of phosphorus (OH on Fe or Ni, H on P, Figure 4d).

**Figure 5:**
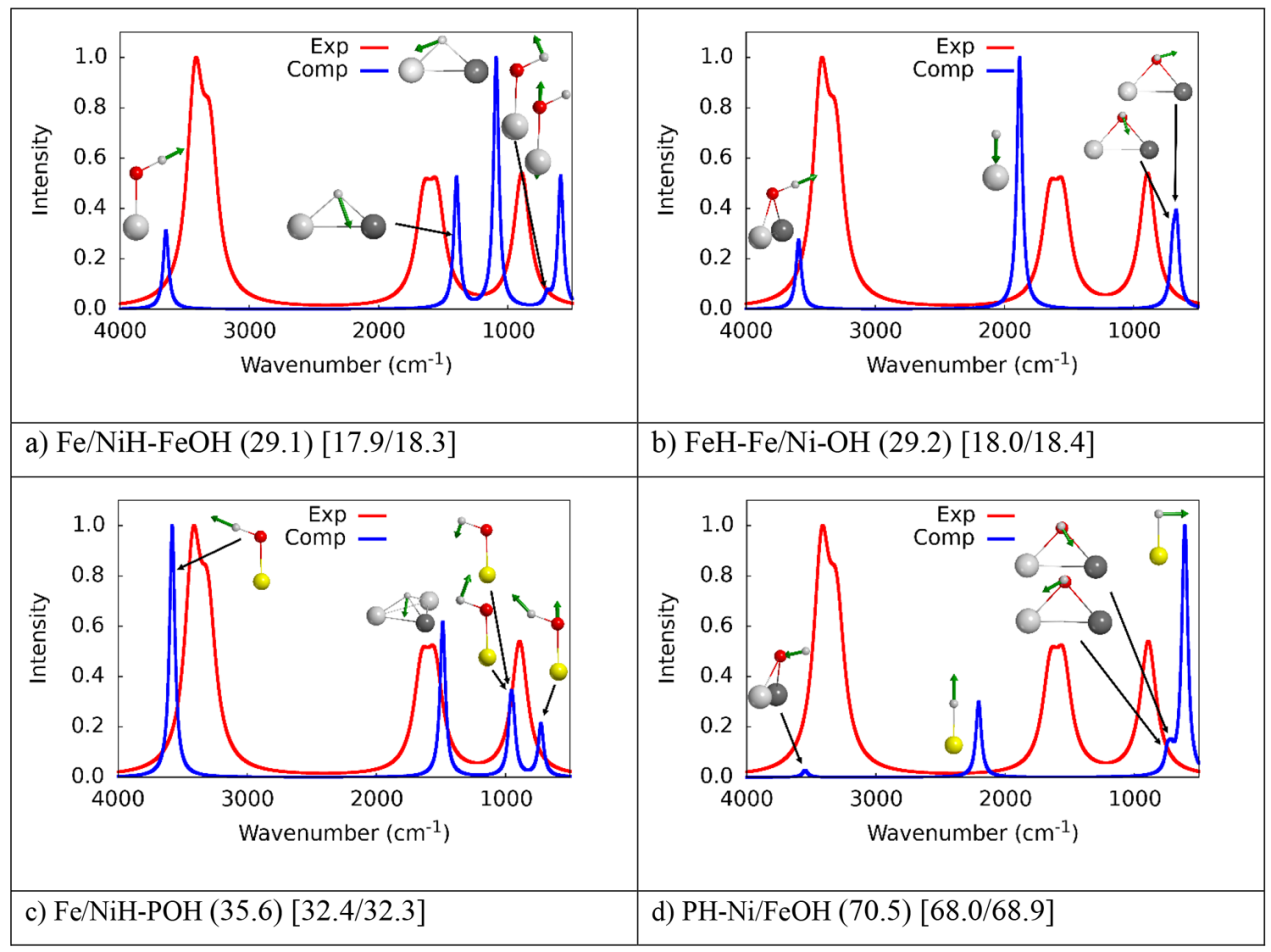
IR spectra of adsorbed deprotonated water on (110) schreibersite. Green arrows represent the motion of the vibrational modes. Atoms color legend: H is in white, O is in red, P is in yellow, Fe is in light gray, and Ni is in dark gray. The experimental spectrum (1.0 Langmuir, 125 K recorded T) is adapted with permission from ref (17). Copyright 2017 ACS.

In contrast to the adsorption of molecular water, the deprotonation leads to a strong electronic rearrangement of the surface due to the proton attachment, which reduces to a hydride ion, with an average charge of −0.27 |e|. Figure 4a and b show the water dissociation at Fe and Ni to have the same DE (around 29 kJ/mol). In contrast, the formation of the P–H bonds destabilizes the systems (Figure 4d, DE = 70.5 kJ/mol), while hydroxylation on P leads to moderately stable structures (Figure 4c, DE = 35.6 kJ/mol), especially when considering the remarkable strength of the P–O bond. Therefore, our results show that, at low temperatures, water deprotonation is not thermodynamically favored. As a further confirmation of our findings, Figure 5 shows the computed spectra of all the adsorption modes with deprotonated water (as shown in Figure 4) in comparison with the experimental one for the water monolayer case. As one can see, all the computed vibrational modes of deprotonated water are qualitatively different with respect to the experimental spectrum, thus, indicating that it is unlikely that water undergoes deprotonation at low temperatures on the (110) surface.

This is also confirmed by the experimental spectra of Figure 3, where in the case of the multilayer water adsorption at room temperature (Figure 3b) a new peak rises at 2423 cm^–1^, which is not present in Figure 3a, where the adsorption is studied at low temperature (125 K). As already pointed out, this was originally assigned to P–H/P–OH stretching, and, accordingly, to water deprotonated at the surface. Considering gas phase phosphonic acid (HP(O)OH_2_), the P–H bond vibrates indeed at 2487 cm^–1^,^42^ and it could, in principle, explain the peak at 2423 cm^–1^, while the stretching associated with P–OH group falls at the lower frequency (below 1000 cm^–1^). However, in the case of schreibersite, phosphorus has a completely different chemical environment than the simpler phosphonic acid. According to our results, P–H vibration represented in Figure 4d is located at 2204 cm^–1^ (without anharmonic correction, which would further cause a bathochromic shift of the peak), which is quite different with respect to the experimental interpretation and, accordingly, can be excluded as origin of the observed band. Nonetheless, Figure 3b, shows that the position and relative intensity of the 2432 cm^–1^ band is correctly reproduced by our simulations. A deeper analysis of the structure associated with this particular feature indicates that it belongs to an incipient proton transfer between two water molecules in close contact (O1H1···O2 of the pair is 1.491 Å, O1–H1 1.047 Å, see Figure 1d) with the schreibersite surface, rather than a newly formed covalent bond with the surface. In that configuration, a proton shuttle between the two oxygen atoms causes a very large bathochromic shift of the original OH stretching bands, as the proton is loosely bound vibrating at 2407 cm^–1^. This value is also in agreement with the value of the frequency associate to an OH bond belonging to the H_3_O^+^ moiety formed in an acidic chabazite engaged in a hydrogen bond with a neighbor water molecule.^43^ We are, however, suspicious about the robustness of the value computed for this vibration, as incipient proton transfer are difficult to be correctly treated by the level of theory adopted here. Indeed, the PBE functional underestimates the OH bond strength in H-bonding interactions giving too loosely bound protons and exceedingly large bathochromic OH shifts. The extreme consequence is a biased easier formation of ion pairs (H_3_O^+^/OH^–^) in a liquid water simulation than it should be.^44^ To further study that feature, a PBE-D*0 molecular dynamics simulation at 300 K was performed for 1 ps and the final structure was then reoptimized followed by an harmonic frequency calculation. The above-mentioned feature does not disappear, meaning that, according to PBE-D*0, the formation of such ion pair (H_3_O^+^/OH^–^) is stable, at least within the very short MD simulation (longer ones are too demanding for our computational facilities). To clarify the dependence of the results on the adopted functional, we reoptimize the PBE-D*0 optimized structure with the BLYP-D*0 functional, followed by a harmonic frequency calculation: as expected, the BLYP functional increases the intermolecular H-bond distance with respect to the PBE (O1H1···O2 from 1.491 to 1.562 Å), while the intramolecular OH bonds strength is shortened (O1–H1 decreases from 1.047 to 1.029 Å). As a direct consequence, the peak moves to higher wavenumbers (from 2408 to 2649 cm^–1^) getting closer to the main OH stretching manifold band. As a further check, we also carried out BLYP optimization and frequency calculations on the PH-Ni/FeOH structure (see Figure 4d of the main text). The P–H stretching slightly decreases from 2205 cm^–1^ (PBE-D*0 value) to 2190 cm^–1^ (BLYP-D*0 value), showing that, in contrast to the O–H stretching, the vibration of the P–H moiety is far less affected by the adopted functional, giving confidence to the PBE-D*0 result for that specific band. In conclusion, we cannot exclude the experimental vibrational feature at 2423 cm^–1^ as due to the formation of an incipient H_3_O^+^/OH^–^ ion pair at the schreibersite surface, in agreement with the fact that, at variance with its absence for a water monolayer, water multilayers are needed to stabilize the ion pair at the schreibersite interface. However, it has to be established whether this spectroscopic feature can be accounted for by less fancy chemical situations, when water will be studied in interaction with the most reactive (001) surface with a most probable stabilization of chemisorbed water over the physisorbed state. Indeed, water chemisorption may result in a deep surface corrosion exposing moieties similar, in the spectral character, to the phosphonic acid (HP(O)OH_2_), with the P–H vibrating at a frequency close to that experimentally observed.

## 4 Conclusions

In the present work, the adsorption of water on the most stable (110) surface of schreibersite was modeled by means of periodic calculations using plane waves and the PBE-D*0 approach. We consider different surface water coverage, from single water molecule per unit cell, to monolayer (1 Langmuir) up to a multilayer case. For the single water molecule case, we reached almost zero coverage by enlarging the surface unit cell area. For the multilayer, we fill in the empty space between surface replicas (about 20 Å), reaching a water density close to 1 g/cm^3^.

Adsorption energy shows that water, when molecularly adsorbed, binds preferentially through an O–metal bond to Ni (−37.6 kJ/mol) and less so to Fe (−29.1 kJ/mol) atoms, both sites acting as electron acceptor of the O electron pairs, stabilized by electronic (Ni reach a d^10^ configuration) and electrostatic (Fe brings a partial positive charge) effects, respectively. In contrast, the interaction with phosphorus is through a very weak H-bond, in which water acts as a hydrogen donor. The IR spectra corresponding to the adsorption of one water molecule per unit cell revealed a weak dependence of the OH stretching bands when the water coverage reached the limit of zero coverage. The most relevant change occurred for the adsorption at Fe atom on the original smallest unit cell, where an increased charge transfer from the surface toward the water molecule causes a larger OH stretching bathochromic shift than that for the cases of smaller water coverage. Spectra of the monolayer case is in general good agreement with the experimental one recorded at 125 K. For the multilayer case, the experimental spectrum revealed a prominent band at 2423 cm^–1^, which was attributed by the authors by water chemisorption forming either P–H or P–OH groups. Our simulated spectrum revealed a feature at 2408 cm^–1^ due to the incipient (H_3_O^+^/OH^–^) pair, only present when water multilayer was considered. While we can exclude the P–H/P–OH attribution, we cannot exclude this computed feature to be due to a specific arrangement of the water molecules adopted to simulate the multilayer or to artifacts in the adopted PBE-D*0 functional, which tends to give a too weak OH bond when engaged in a hydrogen bond.

The deprotonation of water on the (110) surface was studied for the case of a single water molecule per unit cell: the process is thermodynamically unfavorable, being endothermic (around 29 kJ/mol) with respect to the most stable molecular adsorption case.

Work is in progress to extend the study to water adsorption on the most reactive (001) face, which will provide a more complete picture of the role of water chemisorption, a more robust rationalization of the 2423 cm^–1^ band in terms of surface attached species containing the P–H feature and paths for the formation of phosphates.

## Supporting Information Available

A figure with more deprotonated structures and corresponding relative energy with respect to the most stable one and the coordinates of the optimized structures (PDF)
The coordinates of the optimized structures in POSCAR format (ZIP)
