# Supplementary material for: Water Interaction with Fe2NiP Schreibersite (110) Surface: a Quantum Mechanical Atomistic Perspective
Source: J Phys Chem C Nanomater Interfaces. 2022 Jan 25;126(4):2243–52. doi: 10.1021/acs.jpcc.1c09947 (PMC8819687; doi:10.1021/acs.jpcc.1c09947)
Supplement: Supplementary file 1 — jp1c09947_si_001.pdf [file jp1c09947_si_001.pdf]

Water Interaction with Fe_2NiP Schreibersite (110) Surface: a Quantum Mechanical Atomistic Perspective

Supporting information

Stefano Pantaleone,[*1,2] Marta Corno,[1] Albert Rimola,[3] Nadia Balucani,[2,4,5] and Piero Ugliengo*[*1]*

[1]Dipartimento di Chimica and Nanostructured Interfaces and Surfaces (NIS) Centre, Università degli Studi di Torino, via P. Giuria 7, I-10125, Torino, Italy

[2]Dipartimento di Chimica, Biologia e Biotecnologie, Università degli Studi di Perugia, Via Elce di Sotto 8, I-06123 Perugia, Italy

[3]Departament de Química, Universitat Autònoma de Barcelona, 08193 Bellaterra, Catalonia, Spain

[4]Osservatorio Astrofisico di Arcetri, Largo E. Fermi 5, I-50125 Firenze, Italy

[5]Université Grenoble Alpes, CNRS, Institut de Planétologie et d'Astrophysique de Grenoble (IPAG), F-38000 Grenoble, France

*Email: piero.ugliengo@unito.it, stefano.pantaleone@unito.it, stefano.pantaleone@unipg.it

Keywords: Meteorites, phosphorous problem, DFT, prebiotic chemistry, water adsorption

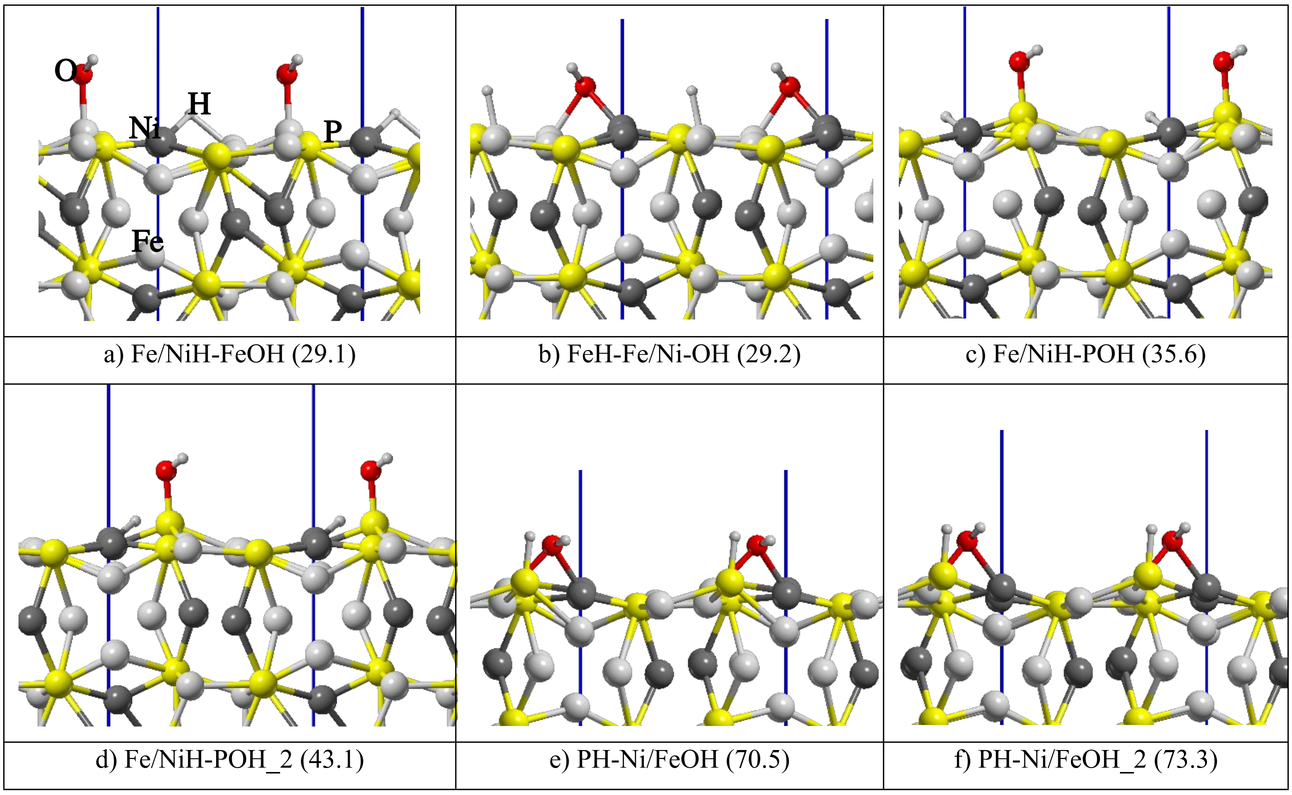

Figure S1: PBE-D*0 optimized structure of deprotonated water molecules adsorbed on the (110) schreibersite surface. H white, O red, P yellow, Fe black, Ni grey. Values in parenthesis are the binding energy per water molecule in kJ/mol.

Optimized structures in POSCAR format

H_2O-Ni

```
H 4   O 2   P 12   Fe 24   Ni 12
  1.000000000
    4.374399     0.000000     0.000000
    -2.187200     6.352773     0.000000
    0.000000     0.000000     40.000000
H   O   P   Fe   Ni
4   2   12   24   12
Cartesian
0.86925998     5.17132999     29.08025000  #H
0.18390999     3.81754000     29.38015000  #H
0.86925999     3.05504000     10.91975000  #H
0.18390999     4.40883000     10.61985000  #H
1.05736999     4.21388000     29.16300000  #O
1.05736999     4.01250000     10.83700000  #O
0.73699998     6.22928999     38.38620000  #P
0.85200999     2.54078000     37.90177000  #P
-1.34444002     5.71357999     35.68258000  #P
2.94794999     2.07937000     35.31145000  #P
-1.24604002     5.83021999     31.65533000  #P
3.16876999     2.27736000     31.43780000  #P
-1.20563002     5.94902999     8.56220000  #P
3.12835999     2.39616000     8.34467000  #P
-1.42645002     6.14701999     4.68855000  #P
3.02995999     2.51281000     4.31742000  #P
0.85200998     5.68559999     2.09823000  #P
0.73699999     1.99709000     1.61380000  #P
-0.39140001     2.93363000     39.84498000  #Fe
1.98456999     4.29242000     38.82216000  #Fe
0.72323000     0.23700000     37.86940000  #Fe
3.05633999     1.99862000     37.54885000  #Fe
0.84563998     6.26055999     36.01580000  #Fe
0.77174000     1.63436000     35.74982000  #Fe
-0.19231001     3.96103000     34.80420000  #Fe
1.81376998     5.24402999     33.68840000  #Fe
1.80491999     2.82847000     33.51038000  #Fe
-0.15051001     4.04999000     32.44024000  #Fe
3.13812000     0.05845000     31.63993000  #Fe
0.99552000     1.65990000     31.51552000  #Fe
3.18272000     0.21372000     8.48448000  #Fe
0.95091999     1.81516000     8.36007000  #Fe
-0.15051001     4.17639000     7.55976000  #Fe
1.80491998     5.39790999     6.48962000  #Fe
1.81376999     2.98236000     6.31160000  #Fe
-0.19231001     4.26535000     5.19580000  #Fe
2.95894000     0.23925000     4.25018000  #Fe
```

0.84564999 1.96581000 3.98420000 #Fe
-1.31807002 6.22774999 2.45115000 #Fe
2.91043000 1.63661000 2.13060000 #Fe
1.98456999 3.93397000 1.17784000 #Fe
-0.39140002 5.29274999 0.15502000 #Fe
1.86137000 1.60418000 39.73578000 #Ni
-0.27063001 4.41288000 37.46455000 #Ni
1.90519999 3.87615000 36.17939000 #Ni
1.78391000 0.19685000 33.74391000 #Ni
-0.38749000 1.53096000 33.48869000 #Ni
1.94741999 4.06296000 31.13747000 #Ni
1.94741999 4.16341000 8.86253000 #Ni
1.79971000 0.34266000 6.51131000 #Ni
-0.40329000 1.67676000 6.25609000 #Ni
1.90519999 4.35023000 3.82061000 #Ni
-0.27063001 3.81350000 2.53545000 #Ni
4.04857000 0.26942000 0.26422000 #Ni

H_2O-Fe

H 4 O 2 P 12 Fe 24 Ni 12
1.000000000
4.374399 0.000000 0.000000
-2.187200 6.352773 0.000000
0.000000 0.000000 40.000000
H O P Fe Ni
4 2 12 24 12
Cartesian
0.87632999 4.20638000 11.13193000 #H
3.06352999 2.14640000 28.86807000 #H
0.01076998 5.41309999 10.69282000 #H
2.19797000 0.93967000 29.30718000 #H
-0.03048001 4.44258000 10.85396000 #O
2.15671999 1.91019000 29.14604000 #O
0.15830999 1.75514000 8.60257000 #P
2.24640999 4.54937000 8.24616000 #P
-0.09428001 2.07360000 4.70410000 #P
2.17008999 4.76568000 4.30270000 #P
2.18466000 1.58206000 2.10819000 #P
-0.12264001 4.24154000 1.60511000 #P
2.06454999 2.11123000 38.39489000 #P
-0.00254001 4.77072000 37.89181000 #P
-0.01711000 1.58709000 35.69730000 #P
2.09291999 4.27918000 35.29590000 #P
0.05920999 1.80340000 31.75384000 #P
2.34550999 4.59763000 31.39743000 #P
2.34929999 2.35065000 8.50617000 #Fe
0.06278999 4.00881000 8.48795000 #Fe
1.20944000 0.00733000 7.54622000 #Fe

3.10187000 1.27512000 6.43416000 #Fe
0.94454998 5.19303999 6.26739000 #Fe
1.13205000 0.16014000 5.17253000 #Fe
2.09143999 2.49854000 4.25642000 #Fe
-0.01547001 4.22729000 3.96236000 #Fe
0.01435999 2.12476000 2.45696000 #Fe
2.05143999 3.88563000 2.12496000 #Fe
1.12577998 6.17807999 1.17655000 #Fe
0.93714000 1.17976000 0.16104000 #Fe
-1.25006002 5.17301999 39.83896000 #Fe
3.31298000 0.17470000 38.82345000 #Fe
-0.13576001 2.46715000 37.87504000 #Fe
2.20155999 4.22801000 37.54304000 #Fe
2.17172999 2.12548000 36.03764000 #Fe
-0.09576001 3.85423000 35.74358000 #Fe
-1.05515002 6.19262999 34.82747000 #Fe
3.13175000 1.15973000 33.73261000 #Fe
0.91466999 5.07764999 33.56584000 #Fe
-0.97776002 6.34543999 32.45378000 #Fe
2.24998999 2.34397000 31.51205000 #Fe
0.16209999 4.00213000 31.49383000 #Fe
1.11387998 6.35264299 8.63482000 #Ni
0.92553999 2.57348000 6.56778000 #Ni
-1.24249001 3.90868000 6.23979000 #Ni
3.23477000 0.26082000 3.81936000 #Ni
-1.13129002 6.06558999 2.52316000 #Ni
3.19126999 2.50955000 0.26955000 #Ni
1.00406999 3.84323000 39.73045000 #Ni
1.05591000 0.28718000 37.47684000 #Ni
1.04756998 6.09194999 36.18064000 #Ni
0.94470999 2.44410000 33.76021000 #Ni
-1.26166001 3.77929000 33.43222000 #Ni
3.30108000 0.00013000 31.36518000 #Ni

H_2O-mono

H 12 O 6 P 12 Fe 24 Ni 12
1.000000000
4.374399 0.000000 0.000000
-2.187200 6.352773 0.000000
0.000000 0.000000 40.000000
H O P Fe Ni
12 6 12 24 12
Cartesian
0.65768398 5.65295699 11.29907400 #H
2.84488400 0.69981600 28.70092600 #H
2.23750198 5.62605299 11.31905200 #H
0.05030300 0.72672000 28.68094800 #H
0.42482999 2.06796100 13.00708500 #H
2.61202999 4.28481200 26.99291500 #H

3.79528800	1.38868500	12.02283500	#H
1.60808899	4.96408900	27.97716500	#H
-0.74648401	4.30616100	10.51216100	#H
1.44071599	2.04661200	29.48783900	#H
-0.63839501	3.73581700	11.96265800	#H
1.54880499	2.61695600	28.03734200	#H
1.46118098	6.21272399	11.08568600	#O
3.64838100	0.14004900	28.91431400	#O
-0.46252501	2.17623800	12.62303600	#O
1.72467399	4.17653600	27.37696400	#O
-0.71975701	4.60449900	11.45985700	#O
1.46744299	1.74827400	28.54014300	#O
0.55586899	1.83875800	8.55565400	#P
2.68012699	4.63991000	8.25254300	#P
0.33767999	2.05566900	4.69594900	#P
2.61304699	4.76746300	4.30678700	#P
2.62271500	1.58096600	2.09866400	#P
0.32017799	4.24339700	1.61070100	#P
2.50737799	2.10937600	38.38929900	#P
0.43551599	4.77180800	37.90133600	#P
0.42584700	1.58531000	35.69321300	#P
2.52487999	4.29710500	35.30405100	#P
0.49292799	1.71286300	31.74745700	#P
2.74306899	4.51401600	31.44434600	#P
2.75604799	2.45671200	8.49618600	#Fe
0.50477999	4.06757900	8.34980700	#Fe
1.64161900	0.07616000	7.57225500	#Fe
3.58341000	1.29219900	6.50553500	#Fe
1.39722098	5.24121899	6.29203500	#Fe
1.57767900	0.16870300	5.19483500	#Fe
2.53500799	2.48694100	4.24703100	#Fe
0.43040599	4.22056300	3.97159700	#Fe
0.45184499	2.12213700	2.45256400	#Fe
2.49459399	3.88410100	2.12775800	#Fe
1.56764898	6.17920599	1.17801700	#Fe
1.37838000	1.17959600	0.15690100	#Fe
-0.80882002	5.17317799	39.84309900	#Fe
3.75484900	0.17356800	38.82198300	#Fe
0.30739399	2.46867200	37.87224200	#Fe
2.63904399	4.23063600	37.54743600	#Fe
2.61760599	2.13221100	36.02840300	#Fe
0.34780799	3.86583300	35.75296900	#Fe
-0.60952102	6.18406999	34.80516500	#Fe
3.58442000	1.11155400	33.70796500	#Fe
1.39621099	5.06057399	33.49446500	#Fe
-0.54558102	6.27661399	32.42774500	#Fe
2.69197899	2.28519400	31.65019300	#Fe
0.56884799	3.89606100	31.50381400	#Fe
3.71295500	0.04695500	8.89162700	#Ni
1.38923999	2.59393000	6.51512400	#Ni

-0.81227601 3.91767900 6.22526400 #Ni
3.67399700 0.25371700 3.82432200 #Ni
-0.68913802 6.06378099 2.53383200 #Ni
-0.74223901 2.51082400 0.26470900 #Ni
1.44496099 3.84195000 39.73529100 #Ni
1.49806100 0.28899200 37.46616800 #Ni
1.48679798 6.09905699 36.17567800 #Ni
1.37492399 2.43509400 33.77473600 #Ni
-0.79796001 3.75884300 33.48487600 #Ni
1.52575398 6.30581799 31.10837300 #Ni

H_2O-Ni-multi

H 38 O 19 P 12 Fe 24 Ni 12
1.000000000
4.361536 0.000000 -0.106245
-2.188342 6.337633 -0.085901
0.000000 0.000000 38.736014
H O P Fe Ni
38 19 12 24 12
Cartesian
0.70239504 4.74486630 24.61231610 #H
-0.17709625 5.91664842 23.93259747 #H
-0.15635932 2.01958554 18.21768067 #H
3.34175171 2.88032799 17.11251755 #H
0.07062041 5.04917435 20.01356949 #H
-0.78530108 4.20446206 18.97687019 #H
0.59611342 0.86618278 22.93371264 #H
4.25721393 0.04769795 23.99497411 #H
-0.99663558 4.01265721 15.14918869 #H
2.41787964 2.71281534 15.06856702 #H
-0.31559592 1.73938946 21.27097563 #H
0.16656848 0.22445953 21.09191020 #H
0.38098887 5.72673124 14.90860413 #H
-0.16349986 5.43003550 13.42840880 #H
0.24272471 2.21889272 15.03599500 #H
1.15999759 0.94900613 14.56147761 #H
1.36566621 0.10311127 18.60046397 #H
2.00720904 1.52214690 18.10574107 #H
2.72358425 2.71182675 22.43743007 #H
1.92081733 2.06306293 21.25086477 #H
1.04340600 5.05721590 22.11593403 #H
1.95857724 4.30322723 21.07580424 #H
4.26198450 0.08736587 16.67978203 #H
2.71757718 0.03667202 16.95609313 #H
-0.65485875 1.99868106 24.41061045 #H
2.14911473 1.79135454 24.33080458 #H
0.96857807 5.70410776 11.08872339 #H
2.66227053 0.28552783 27.30573336 #H
-1.80957633 5.71150416 11.18766692 #H

4.24341264	0.33276563	27.26828084	#H
0.19136914	2.21882080	13.05945466	#H
2.08940566	3.56522468	25.54434337	#H
-0.46008977	1.59247273	11.75782409	#H
1.33920778	4.55274138	26.59419738	#H
-0.42924057	4.51999108	10.38125788	#H
1.27763641	1.62009483	28.15029535	#H
-0.42433221	3.76854214	11.76917352	#H
1.14110463	2.20358416	26.71085218	#H
0.67348886	5.40326020	23.86261980	#O
3.39909971	2.59927355	18.07585783	#O
-0.71447690	5.09557835	19.40942567	#O
0.64860350	0.69449681	23.93940140	#O
3.25349453	3.11776655	15.45440158	#O
0.48776623	1.13734340	21.34281557	#O
-0.44469265	5.58317697	14.35009941	#O
1.04789702	1.93453312	14.50997418	#O
1.27297038	0.86420375	17.93514429	#O
2.68850310	2.67272568	21.44638399	#O
1.36805410	5.09333647	21.18265142	#O
1.24008637	5.99643317	16.30052666	#O
2.84648837	2.48492270	24.25425222	#O
1.75515220	6.25936346	10.81194430	#O
1.28166055	6.07426846	27.42086754	#O
-0.47726466	2.39190113	12.33829535	#O
1.21952969	3.78319171	25.96304303	#O
-0.41372420	4.72857376	11.35130054	#O
1.22867396	1.33265908	27.19586263	#O
0.84134534	1.82024614	8.46018445	#P
-1.41187283	4.60820635	8.14670626	#P
0.53677377	1.96575016	4.60148410	#P
-1.56685337	4.66098399	4.20203804	#P
2.75775032	1.44170763	1.95520174	#P
0.44784318	4.08539678	1.46403764	#P
2.55801838	1.89961166	36.97642210	#P
0.47840176	4.54344036	36.47902292	#P
0.42177185	1.32341866	34.33994071	#P
2.50242375	4.01555237	33.83908576	#P
0.39521338	1.37147090	30.39848077	#P
2.63428210	4.15873715	29.95976139	#P
3.03226782	2.43461033	8.33430814	#Fe
0.78348906	4.04019394	8.20495990	#Fe
1.89684053	0.04323796	7.48050172	#Fe
3.81910035	1.23666285	6.34971339	#Fe
1.63055354	5.17291991	6.10039916	#Fe
1.78925266	0.09196813	5.10876282	#Fe
2.71629925	2.38425139	4.08659239	#Fe
0.60982523	4.11046630	3.82127488	#Fe
0.60002638	1.98537725	2.35373025	#Fe
2.62794683	3.73863815	1.94052483	#Fe

1.67907466   6.01050817   0.95910497 #Fe
1.47545312   1.00371098   0.05314074 #Fe
-0.71670353   4.98193093   38.44076705 #Fe
1.62620214   6.31301815   37.33426486 #Fe
0.35118740   2.24547372   36.50189516 #Fe
2.66658206   3.99830466   36.08075496 #Fe
2.61388691   1.87240996   34.61535917 #Fe
0.34233910   3.59890413   34.35481681 #Fe
-0.63660274   5.89219324   33.37861849 #Fe
3.52768158   0.81124909   32.29460537 #Fe
1.33706298   4.74725586   32.04271917 #Fe
-0.62938331   5.93821649   31.00097199 #Fe
2.58507656   1.94084559   30.23016652 #Fe
0.46596893   3.54280449   30.09508753 #Fe
3.99247673   0.03813565   8.76246943 #Ni
1.63128372   2.53741238   6.38380637 #Ni
-0.57087020   3.85030091   6.11188751 #Ni
3.84723150   0.14851813   3.68397679 #Ni
-0.53937191   5.92273361   2.37395080 #Ni
-0.63642493   2.33407292   0.18513766 #Ni
1.53074058   3.65236032   38.30764770 #Ni
1.53308004   0.06237674   36.11512202 #Ni
1.48680275   5.83499936   34.70093363 #Ni
1.32843386   2.13640179   32.38483340 #Ni
-0.84784143   3.45108897   32.11029456 #Ni
1.40200634   5.93830419   29.63946663 #Ni

H_2O-Ni_2x1

H 2   O 1   P 24   Fe 48   Ni 24
1.000000000
8.748798   0.000000   0.000000
-2.187200   6.352773   0.000000
0.000000   0.000000   40.000000
H   O   P   Fe   Ni
2   1   24   48   24
Cartesian
0.91868999   3.09588000   11.01956000 #H
0.14351999   4.38256000   10.63377000 #H
1.04674999   4.04621000   10.82713000 #O
0.73460998   6.22564999   38.38395000 #P
0.85170999   2.53616000   37.89541000 #P
-1.34621002   5.70703999   35.68088000 #P
2.95313999   2.07022000   35.30873000 #P
-1.22166002   5.83094999   31.60580000 #P
3.18850999   2.28745000   31.42219000 #P
-1.18329002   5.93467999   8.59001000 #P
3.11658999   2.40951000   8.35307000 #P
-1.42070002   6.15146999   4.67773000 #P
3.03106999   2.51470000   4.32349000 #P

0.85435998	5.68401999	2.09977000	#P
0.73817999	1.99655000	1.61097000	#P
5.10870998	6.22563999	38.38238000	#P
5.22561999	2.53628000	37.89457000	#P
3.02794998	5.70705999	35.68075000	#P
7.32742999	2.07020000	35.30838000	#P
3.15263998	5.83093999	31.60601000	#P
7.56298999	2.28738000	31.42208000	#P
3.15385998	5.96349999	8.55023000	#P
7.52297999	2.39054000	8.39460000	#P
2.95234998	6.14837999	4.69394000	#P
7.40647999	2.51761000	4.30412000	#P
5.22701998	5.68602999	2.09805000	#P
5.11093999	1.99595000	1.61068000	#P
-0.39162001	2.93052000	39.84110000	#Fe
1.98371999	4.28901000	38.82028000	#Fe
0.72072000	0.23288000	37.86731000	#Fe
3.05545999	1.99202000	37.54470000	#Fe
0.84212998	6.25326999	36.00546000	#Fe
0.77285000	1.62125000	35.74055000	#Fe
-0.20058001	3.95834000	34.80755000	#Fe
1.79904998	5.25417999	33.70109000	#Fe
1.77868999	2.80941000	33.54423000	#Fe
-0.12780001	4.05133000	32.42896000	#Fe
3.15404000	0.04816000	31.59681000	#Fe
1.01189000	1.67741000	31.50141000	#Fe
3.18485000	0.22942000	8.48583000	#Fe
0.95084999	1.79811000	8.37097000	#Fe
-0.10535001	4.16461000	7.54241000	#Fe
1.80174998	5.40962999	6.49375000	#Fe
1.81041999	2.97910000	6.31757000	#Fe
-0.19628001	4.26818000	5.18734000	#Fe
2.96295000	0.24331000	4.25489000	#Fe
0.84825999	1.96805000	3.98805000	#Fe
-1.31548002	6.23023999	2.44494000	#Fe
2.91205000	1.63667000	2.13071000	#Fe
1.98707999	3.93191000	1.17725000	#Fe
-0.39151002	5.29010999	0.15262000	#Fe
3.98323999	2.93175000	39.84194000	#Fe
6.35793999	4.28909000	38.81887000	#Fe
5.09416000	0.23250000	37.86829000	#Fe
7.43005999	1.99237000	37.54402000	#Fe
5.21650998	6.25329999	36.00507000	#Fe
5.14718000	1.62097000	35.74097000	#Fe
4.17371999	3.95837000	34.80746000	#Fe
6.17349998	5.25416999	33.70097000	#Fe
6.15302999	2.80939000	33.54413000	#Fe
4.24656999	4.05137000	32.42897000	#Fe
7.52849000	0.04806000	31.59669000	#Fe
5.38628000	1.67748000	31.50138000	#Fe

7.55918000 0.18564000 8.49647000 #Fe
5.32683999 1.85434000 8.39107000 #Fe
4.19461999 4.19322000 7.59381000 #Fe
6.15114998 5.41752999 6.44578000 #Fe
6.17657999 2.97956000 6.28642000 #Fe
4.17962999 4.26668000 5.20204000 #Fe
7.33505000 0.24923000 4.25232000 #Fe
5.21927999 1.96707000 3.99102000 #Fe
3.05690998 6.22941999 2.45399000 #Fe
7.28400000 1.63515000 2.12426000 #Fe
6.35940999 3.93243000 1.17465000 #Fe
3.98274998 5.29113999 0.15388000 #Fe
1.86152000 1.60226000 39.73283000 #Ni
-0.27215001 4.40899000 37.46197000 #Ni
1.89977999 3.86808000 36.17542000 #Ni
1.77061000 0.17956000 33.71728000 #Ni
-0.40182000 1.52787000 33.46720000 #Ni
1.95241999 4.06201000 31.29740000 #Ni
1.93369999 4.17073000 8.90149000 #Ni
1.78690000 0.33379000 6.51883000 #Ni
-0.41544000 1.69943000 6.26774000 #Ni
1.90721999 4.35251000 3.82378000 #Ni
-0.26864001 3.81142000 2.53148000 #Ni
4.04842000 0.26651000 0.26378000 #Ni
6.23473000 1.60311000 39.73085000 #Ni
4.10173999 4.40922000 37.46168000 #Ni
6.27412999 3.86808000 36.17487000 #Ni
6.14499000 0.17967000 33.71719000 #Ni
3.97254000 1.52787000 33.46740000 #Ni
6.32685999 4.06201000 31.29733000 #Ni
6.33169999 4.16822000 8.66993000 #Ni
6.16648000 0.36310000 6.52154000 #Ni
3.96434000 1.68383000 6.27175000 #Ni
6.27635999 4.35512000 3.81763000 #Ni
4.10531999 3.81323000 2.53844000 #Ni
8.42228000 0.26717000 0.25994000 #Ni

H_2O-Ni_2x2

H 2 O 1 P 48 Fe 96 Ni 48
1.000000000
8.748798 0.000000 0.000000
-4.374400 12.705546 0.000000
0.000000 0.000000 40.000000
H O P Fe Ni
2 1 48 96 48
Cartesian
0.92462999 3.07278000 11.01877000 #H
0.17382999 4.38658000 10.67410000 #H
1.07201999 4.02034000 10.82618000 #O

0.73345998	6.22541999	38.38636000	#P
0.85091999	2.53558000	37.89632000	#P
-1.34772002	5.70768999	35.68312000	#P
2.95143999	2.07105000	35.31002000	#P
-1.22535002	5.83252999	31.61044000	#P
3.18626999	2.28845000	31.42245000	#P
-1.17990002	5.94029999	8.59303000	#P
3.12012999	2.38929000	8.34543000	#P
-1.42144002	6.15920999	4.68633000	#P
3.02996999	2.51920000	4.32007000	#P
0.85249998	5.68690999	2.10505000	#P
0.73643999	1.99996000	1.61099000	#P
-1.45319004	12.57781999	38.38347000	#P
-1.33636003	8.88781999	37.89686000	#P
-3.53504004	12.06054999	35.68209000	#P
0.76429998	8.42373999	35.31059000	#P
-3.41249004	12.18528999	31.61022000	#P
0.99871997	8.64098999	31.42345000	#P
-3.37485004	12.28290999	8.57213000	#P
0.96586997	8.76477999	8.39732000	#P
-3.60701004	12.50318999	4.67941000	#P
0.84241997	8.87102999	4.31736000	#P
-1.33278004	12.04033999	2.09709000	#P
-1.45186002	8.35078999	1.61125000	#P
5.10766998	6.22531999	38.38497000	#P
5.22519999	2.53561000	37.89590000	#P
3.02650998	5.70781999	35.68288000	#P
7.32581999	2.07099000	35.30991000	#P
3.14901998	5.83252999	31.61043000	#P
7.56064999	2.28842000	31.42241000	#P
3.16181998	5.95167999	8.55303000	#P
7.53052999	2.39291000	8.40367000	#P
2.95068998	6.14835999	4.69597000	#P
7.40697999	2.52240000	4.30480000	#P
5.22660998	5.68974999	2.10031000	#P
5.11027999	2.00061000	1.61018000	#P
2.92099996	12.57748999	38.38307000	#P
3.03766997	8.88794999	37.89660000	#P
0.83927996	12.06057999	35.68199000	#P
5.13860998	8.42369999	35.31047000	#P
0.96187996	12.18527999	31.61024000	#P
5.37309997	8.64098999	31.42335000	#P
0.99403996	12.29963999	8.57393000	#P
5.33382997	8.74177999	8.37636000	#P
0.76865996	12.51257999	4.68647000	#P
5.21550997	8.86961999	4.31453000	#P
3.04004996	12.04003999	2.09961000	#P
2.92126998	8.34963999	1.61179000	#P
-0.39316001	2.93257000	39.84143000	#Fe
1.98300999	4.28970000	38.82198000	#Fe

0.72014000	0.23219000	37.86792000	#Fe
3.05491999	1.99137000	37.54591000	#Fe
0.84061998	6.25356999	36.00736000	#Fe
0.77127000	1.62252000	35.74170000	#Fe
-0.20173001	3.95942000	34.80929000	#Fe
1.79723998	5.25554999	33.70346000	#Fe
1.77656999	2.81109000	33.54541000	#Fe
-0.12968001	4.05261000	32.42991000	#Fe
3.15093000	0.04882000	31.59873000	#Fe
1.00939000	1.67859000	31.50221000	#Fe
3.19330000	0.19256000	8.48491000	#Fe
0.95690999	1.79308000	8.37085000	#Fe
-0.10075001	4.16658000	7.54955000	#Fe
1.80352998	5.41361999	6.50091000	#Fe
1.81131999	2.97959000	6.31707000	#Fe
-0.19767001	4.27146000	5.19315000	#Fe
2.96207000	0.24864000	4.24672000	#Fe
0.84681999	1.96963000	3.99157000	#Fe
-1.31660002	6.23297999	2.45034000	#Fe
2.91073000	1.64270000	2.12699000	#Fe
1.98427999	3.93536000	1.17845000	#Fe
-0.39300002	5.29197999	0.15617000	#Fe
-2.57984003	9.28483999	39.84193000	#Fe
-0.20390003	10.64167999	38.82066000	#Fe
-1.46761002	6.58519999	37.86927000	#Fe
0.86730998	8.34378999	37.54641000	#Fe
-1.34660004	12.60647999	36.00651000	#Fe
-1.41587002	7.97500999	35.74238000	#Fe
-2.38881003	10.31200999	34.80909000	#Fe
-0.38998003	11.60837999	33.70305000	#Fe
-0.41049003	9.16374999	33.54586000	#Fe
-2.31693003	10.40527999	32.42988000	#Fe
0.96362998	6.40145999	31.59920000	#Fe
-1.17815002	8.03135999	31.50274000	#Fe
1.00529998	6.58342999	8.49619000	#Fe
-1.22381002	8.18167999	8.39854000	#Fe
-2.31923003	10.52350999	7.55776000	#Fe
-0.40817003	11.76408999	6.44429000	#Fe
-0.38684003	9.32220999	6.29511000	#Fe
-2.38538003	10.61861999	5.18162000	#Fe
0.77404998	6.60182999	4.26441000	#Fe
-1.34269002	8.32677999	3.98681000	#Fe
-3.50296004	12.58675999	2.44588000	#Fe
0.72195998	7.98924999	2.13267000	#Fe
-0.20175003	10.28544999	1.17640000	#Fe
-2.57978003	11.64438999	0.15268000	#Fe
3.98190999	2.93363000	39.84169000	#Fe
6.35730999	4.28968000	38.82126000	#Fe
5.09408000	0.23196000	37.86817000	#Fe
7.42933999	1.99133000	37.54581000	#Fe

5.21506998	6.25357999	36.00698000	#Fe
5.14564000	1.62243000	35.74185000	#Fe
4.17256999	3.95948000	34.80913000	#Fe
6.17170998	5.25559999	33.70339000	#Fe
6.15099999	2.81110000	33.54534000	#Fe
4.24469999	4.05265000	32.42989000	#Fe
7.52534000	0.04881000	31.59872000	#Fe
5.38377000	1.67858000	31.50209000	#Fe
7.56879000	0.19630000	8.50151000	#Fe
5.32996999	1.84154000	8.39344000	#Fe
4.19472999	4.18121000	7.59277000	#Fe
6.14904998	5.41013999	6.44443000	#Fe
6.17624999	2.97074000	6.28934000	#Fe
4.17875999	4.26793000	5.20153000	#Fe
7.33607000	0.25316000	4.25599000	#Fe
5.22034999	1.97445000	3.98369000	#Fe
3.05584998	6.23259999	2.45684000	#Fe
7.28465000	1.63748000	2.12466000	#Fe
6.35831999	3.93510000	1.17719000	#Fe
3.98145998	5.29273999	0.15621000	#Fe
1.79440997	9.28490999	39.84248000	#Fe
4.17030997	10.64176999	38.82004000	#Fe
2.90615998	6.58490999	37.86986000	#Fe
5.24187998	8.34400999	37.54612000	#Fe
3.02780996	12.60646999	36.00647000	#Fe
2.95848998	7.97482999	35.74256000	#Fe
1.98555997	10.31207999	34.80910000	#Fe
3.98444997	11.60837999	33.70307000	#Fe
3.96384997	9.16375999	33.54581000	#Fe
2.05750997	10.40530999	32.42991000	#Fe
5.33804998	6.40145999	31.59912000	#Fe
3.19625998	8.03132999	31.50277000	#Fe
5.37844998	6.53469999	8.49068000	#Fe
3.15315998	8.18794999	8.39742000	#Fe
2.06147997	10.53648999	7.56731000	#Fe
3.96928997	11.77505999	6.45372000	#Fe
3.98852997	9.33254999	6.29109000	#Fe
1.98886997	10.62082999	5.18834000	#Fe
5.14693998	6.60257999	4.25227000	#Fe
3.02919998	8.32121999	3.99709000	#Fe
0.87122996	12.58668999	2.44935000	#Fe
5.09426998	7.99304999	2.12820000	#Fe
4.17082997	10.28628999	1.17503000	#Fe
1.79466997	11.64452999	0.15366000	#Fe
1.85944000	1.60337000	39.73277000	#Ni
-0.27271001	4.40867000	37.46444000	#Ni
1.89885999	3.86872000	36.17766000	#Ni
1.76890000	0.18177000	33.71948000	#Ni
-0.40376000	1.53060000	33.46786000	#Ni
1.95035999	4.06328000	31.29862000	#Ni

1.93913999	4.15936000	8.90211000	#Ni
1.78939000	0.34780000	6.51326000	#Ni
-0.41885000	1.70066000	6.27259000	#Ni
1.90340999	4.35633000	3.82632000	#Ni
-0.27037001	3.81398000	2.53397000	#Ni
4.04671000	0.26808000	0.26246000	#Ni
-0.32701002	7.95543999	39.73437000	#Ni
-2.45964003	10.76081999	37.46334000	#Ni
-0.28814003	10.22145999	36.17734000	#Ni
-0.41830002	6.53440999	33.72003000	#Ni
-2.59090002	7.88309999	33.46862000	#Ni
-0.23685003	10.41592999	31.29889000	#Ni
-0.24020003	10.52270999	8.69514000	#Ni
-0.40271002	6.68107999	6.53587000	#Ni
-2.59865002	8.04266999	6.27248000	#Ni
-0.28231003	10.71045999	3.81842000	#Ni
-2.45771003	10.16890999	2.53065000	#Ni
1.85971998	6.61909999	0.26643000	#Ni
6.23348000	1.60288000	39.73211000	#Ni
4.10152999	4.40873000	37.46386000	#Ni
6.27326999	3.86860000	36.17730000	#Ni
6.14328000	0.18182000	33.71944000	#Ni
3.97062000	1.53061000	33.46790000	#Ni
6.32477999	4.06328000	31.29858000	#Ni
6.33353999	4.16605000	8.66652000	#Ni
6.16010000	0.34282000	6.53221000	#Ni
3.96864000	1.68578000	6.26347000	#Ni
6.27671999	4.35960000	3.82037000	#Ni
4.10275999	3.81858000	2.53763000	#Ni
8.42101000	0.26744000	0.26088000	#Ni
4.04623998	7.95630999	39.73318000	#Ni
1.91443997	10.76080999	37.46328000	#Ni
4.08623997	10.22143999	36.17719000	#Ni
3.95604998	6.53447999	33.71990000	#Ni
1.78351998	7.88310999	33.46871000	#Ni
4.13753997	10.41596999	31.29881000	#Ni
4.13928997	10.51522999	8.70390000	#Ni
3.98015998	6.71708999	6.52046000	#Ni
1.76849998	8.04444999	6.28548000	#Ni
4.08837997	10.70882999	3.81699000	#Ni
1.91715997	10.16663999	2.53386000	#Ni
6.23352998	6.62106999	0.26364000	#Ni

H_2O-Fe_2x1

H 2 O 1 P 24 Fe 48 Ni 24
1.000000000

8.748798	0.000000	0.000000
-2.187200	6.352773	0.000000
0.000000	0.000000	40.000000

H O P Fe Ni
2 1 24 48 24
Cartesian
0.90771999 4.41081000 11.12042000 #H
-0.28590002 5.35242999 10.82753000 #H
-0.02600001 4.40783000 10.83457000 #O
0.14086999 1.78996000 8.58770000 #P
2.28299999 4.59546000 8.31431000 #P
-0.09052001 2.05836000 4.70497000 #P
2.16639999 4.76263000 4.31005000 #P
2.18133000 1.58116000 2.10534000 #P
-0.12321001 4.24300000 1.61231000 #P
2.06641999 2.11531000 38.38828000 #P
-0.00189001 4.77853000 37.89732000 #P
-0.01288000 1.59532000 35.68502000 #P
2.10090999 4.31011000 35.31140000 #P
0.11445999 1.72493000 31.61161000 #P
2.33768999 4.53368000 31.42360000 #P
4.52989999 1.80148000 8.59263000 #P
6.62957999 4.59819000 8.31020000 #P
4.28028999 2.05442000 4.68789000 #P
6.54597999 4.76129000 4.31610000 #P
6.55784000 1.57963000 2.10588000 #P
4.25123999 4.24192000 1.60971000 #P
6.44127999 2.11549000 38.38858000 #P
4.37266999 4.77850000 37.89721000 #P
4.36156000 1.59531000 35.68493000 #P
6.47532999 4.31021000 35.31140000 #P
4.48901999 1.72484000 31.61137000 #P
6.71200999 4.53369000 31.42373000 #P
2.34801999 2.39439000 8.50530000 #Fe
0.08379999 4.04655000 8.50357000 #Fe
1.19609000 0.04252000 7.56752000 #Fe
3.09798000 1.29512000 6.43855000 #Fe
0.94150998 5.20421999 6.29443000 #Fe
1.13057000 0.15673000 5.19179000 #Fe
2.09294999 2.49640000 4.25909000 #Fe
-0.01714001 4.21980000 3.98363000 #Fe
0.01084999 2.12398000 2.45902000 #Fe
2.04925999 3.88346000 2.12789000 #Fe
1.12556998 6.17974999 1.17765000 #Fe
0.93792000 1.18373000 0.15839000 #Fe
-1.24907002 5.17669999 39.84363000 #Fe
3.31635000 0.18046000 38.82218000 #Fe
-0.13437001 2.47398000 37.87181000 #Fe
2.20279999 4.23350000 37.54813000 #Fe
2.17556999 2.14116000 36.01037000 #Fe
-0.07973001 3.86221000 35.74413000 #Fe
-1.05312002 6.20057999 34.81168000 #Fe
3.13472000 1.14587000 33.70548000 #Fe

0.92747999	5.05385999	33.54727000	#Fe
-0.97842002	6.29720999	32.43249000	#Fe
2.30325999	2.29427000	31.60002000	#Fe
0.16085999	3.92361000	31.50365000	#Fe
6.71197999	2.40218000	8.49678000	#Fe
4.46139999	4.03386000	8.39217000	#Fe
5.59096000	0.03827000	7.55157000	#Fe
7.48639000	1.28705000	6.45353000	#Fe
5.31713998	5.20609999	6.27801000	#Fe
5.50499000	0.15719000	5.17527000	#Fe
6.47683999	2.49410000	4.25775000	#Fe
4.35950999	4.21926000	3.97696000	#Fe
4.38748999	2.12321000	2.45173000	#Fe
6.42532999	3.88460000	2.13002000	#Fe
5.50070998	6.17885999	1.17846000	#Fe
5.31283000	1.18315000	0.15708000	#Fe
3.12511998	5.17631999	39.84282000	#Fe
7.69093000	0.18030000	38.82285000	#Fe
4.24023999	2.47408000	37.87121000	#Fe
6.57737999	4.23346000	37.54807000	#Fe
6.55001999	2.14122000	36.01027000	#Fe
4.29469999	3.86225000	35.74388000	#Fe
3.32126998	6.20050999	34.81163000	#Fe
7.50916000	1.14578000	33.70546000	#Fe
5.30187999	5.05381999	33.54717000	#Fe
3.39604998	6.29720999	32.43248000	#Fe
6.67767999	2.29434000	31.59995000	#Fe
4.53522999	3.92354000	31.50356000	#Fe
3.30466000	0.03962000	8.64654000	#Ni
0.91864999	2.59546000	6.56324000	#Ni
-1.25559001	3.92704000	6.27743000	#Ni
3.22881000	0.25253000	3.82069000	#Ni
-1.13062002	6.06267999	2.53279000	#Ni
3.19000999	2.51222000	0.26617000	#Ni
1.00294999	3.84684000	39.73443000	#Ni
1.06126000	0.29793000	37.46620000	#Ni
1.04701998	6.10849999	36.17901000	#Ni
0.92003999	2.42413000	33.72198000	#Ni
-1.25267001	3.77373000	33.46904000	#Ni
1.10200998	6.30872999	31.30130000	#Ni
7.65202000	0.01883000	8.70429000	#Ni
5.30497999	2.56850000	6.53375000	#Ni
3.11048999	3.91833000	6.24873000	#Ni
7.60923000	0.25219000	3.82329000	#Ni
3.24429998	6.06130999	2.52967000	#Ni
7.56497999	2.51193000	0.26827000	#Ni
5.37829999	3.84641000	39.73422000	#Ni
5.43587000	0.29794000	37.46606000	#Ni
5.42141998	6.10856999	36.17929000	#Ni
5.29438999	2.42405000	33.72183000	#Ni

3.12169999   3.77380000   33.46896000 #Ni
5.47636998   6.30873999   31.30107000 #Ni

H_2O-Fe_2x2

H 2   O 1   P 48   Fe 96   Ni 48
1.000000000
8.748798   0.000000   0.000000
-4.374400   12.705546   0.000000
0.000000   0.000000   40.000000
H   O   P   Fe   Ni
2   1   48   96   48
Cartesian
-1.30286003   10.60564999   11.15446000 #H
-2.33848003   11.71672999   10.84185000 #H
-2.21177003   10.74542999   10.82657000 #O
0.14288999   1.79772000   8.57674000 #P
2.29308999   4.60320000   8.38495000 #P
-0.09041001   2.03111000   4.68614000 #P
2.16812999   4.74949000   4.31636000 #P
2.18017000   1.57356000   2.09828000 #P
-0.12459001   4.23792000   1.61396000 #P
2.06412999   2.12127000   38.38536000 #P
-0.00537001   4.78495000   37.89903000 #P
-0.01591000   1.60672000   35.68158000 #P
2.09750999   4.32270000   35.31109000 #P
0.11185999   1.73950000   31.60921000 #P
2.33474999   4.54837000   31.42412000 #P
-2.04622002   8.14086999   8.58591000 #P
0.09710997   10.95093999   8.32097000 #P
-2.28213002   8.39678999   4.70698000 #P
-0.02432003   11.10074999   4.31645000 #P
-0.00985002   7.92547999   2.10773000 #P
-2.31260003   10.58569999   1.61236000 #P
-0.12367002   8.47533999   38.38931000 #P
-2.19263003   11.13694999   37.89692000 #P
-2.20341002   7.95947999   35.68396000 #P
-0.08988003   10.67383999   35.30909000 #P
-2.07560002   8.09250999   31.61139000 #P
0.14757997   10.90090999   31.42190000 #P
4.53253999   1.80487000   8.58570000 #P
6.67110999   4.60807000   8.39201000 #P
4.28400999   2.03601000   4.68275000 #P
6.54330999   4.75022000   4.31829000 #P
6.55495000   1.57243000   2.09912000 #P
4.25057999   4.23736000   1.61330000 #P
6.43865999   2.12122000   38.38561000 #P
4.36916999   4.78487000   37.89902000 #P
4.35855000   1.60666000   35.68179000 #P
6.47186999   4.32271000   35.31116000 #P

4.48627999	1.73954000	31.60918000	#P
6.70916999	4.54837000	31.42420000	#P
2.32772998	8.14604999	8.58349000	#P
4.44127997	10.94908999	8.31574000	#P
2.08858998	8.38996999	4.69355000	#P
4.35444997	11.09849999	4.31940000	#P
4.36654998	7.92541999	2.10725000	#P
2.06099997	10.58511999	1.61001000	#P
4.25114998	8.47554999	38.38920000	#P
2.18189997	11.13695999	37.89679000	#P
2.17099998	7.95946999	35.68388000	#P
4.28452997	10.67390999	35.30895000	#P
2.29877998	8.09246999	31.61136000	#P
4.52193997	10.90087999	31.42207000	#P
2.34390999	2.40194000	8.49343000	#Fe
0.10990999	4.03737000	8.40193000	#Fe
1.19597000	0.03887000	7.56497000	#Fe
3.09836000	1.28667000	6.43181000	#Fe
0.94086998	5.19442999	6.29587000	#Fe
1.13167000	0.13888000	5.18597000	#Fe
2.10116999	2.48401000	4.25096000	#Fe
-0.01828001	4.20410000	3.99455000	#Fe
0.00943999	2.11620000	2.45039000	#Fe
2.04768999	3.87790000	2.12766000	#Fe
1.12457998	6.17594999	1.17938000	#Fe
0.93645000	1.18202000	0.15267000	#Fe
-1.25076002	5.17649999	39.84592000	#Fe
3.31424000	0.18337000	38.81891000	#Fe
-0.13741001	2.48067000	37.86869000	#Fe
2.19926999	4.24244000	37.54766000	#Fe
2.17240999	2.15205000	36.00778000	#Fe
-0.08301001	3.87395000	35.74364000	#Fe
-1.05612002	6.21256999	34.81182000	#Fe
3.13134000	1.15873000	33.70305000	#Fe
0.92381999	5.06748999	33.54726000	#Fe
-0.98093002	6.31159999	32.43232000	#Fe
2.30043999	2.30883000	31.59837000	#Fe
0.15799999	3.93835000	31.50300000	#Fe
0.15745997	8.75109999	8.50638000	#Fe
-2.10490003	10.39764999	8.50789000	#Fe
-0.98598002	6.38844999	7.57232000	#Fe
0.91649998	7.63604999	6.45753000	#Fe
-1.24802003	11.54592999	6.29895000	#Fe
-1.06139002	6.49943999	5.19582000	#Fe
-0.09652003	8.83362999	4.26342000	#Fe
-2.20793003	10.55925999	3.98495000	#Fe
-2.18019002	8.46490999	2.46125000	#Fe
-0.14142003	10.22761999	2.13257000	#Fe
-1.06183004	12.52571999	1.17403000	#Fe
-1.25087002	7.53577999	0.15878000	#Fe

-3.43733003	11.52762999	39.84295000	#Fe
1.12665998	6.53748999	38.82333000	#Fe
-2.32574003	8.83337999	37.87069000	#Fe
0.01164997	10.59520999	37.54574000	#Fe
-0.01530002	8.50397999	36.01020000	#Fe
-2.27072003	10.22591999	35.74336000	#Fe
-3.24327004	12.56477999	34.80915000	#Fe
0.94451998	7.51203999	33.70510000	#Fe
-1.26296003	11.42006999	33.54522000	#Fe
-3.16794004	12.66414999	32.42948000	#Fe
0.11291997	8.66143999	31.59893000	#Fe
-2.02912003	10.29112999	31.50088000	#Fe
6.71930999	2.41468000	8.49509000	#Fe
4.48201999	4.03898000	8.39941000	#Fe
5.59408000	0.03829000	7.55256000	#Fe
7.48463000	1.28606000	6.44933000	#Fe
5.31384998	5.19056999	6.29551000	#Fe
5.50534000	0.14275000	5.17532000	#Fe
6.47779999	2.48220000	4.25485000	#Fe
4.35808999	4.20561000	3.98934000	#Fe
4.38477999	2.11547000	2.44827000	#Fe
6.42324999	3.87653000	2.13017000	#Fe
5.49944998	6.17520999	1.17986000	#Fe
5.31097000	1.18170000	0.15228000	#Fe
3.12367998	5.17623999	39.84561000	#Fe
7.68885000	0.18341000	38.81891000	#Fe
4.23708999	2.48083000	37.86857000	#Fe
6.57364999	4.24232000	37.54774000	#Fe
6.54680999	2.15207000	36.00784000	#Fe
4.29135999	3.87390000	35.74355000	#Fe
3.31834998	6.21256999	34.81172000	#Fe
7.50572000	1.15864000	33.70296000	#Fe
5.29823999	5.06741999	33.54723000	#Fe
3.39344998	6.31158999	32.43223000	#Fe
6.67484999	2.30886000	31.59840000	#Fe
4.53239999	3.93840000	31.50285000	#Fe
4.51926997	8.74723999	8.49960000	#Fe
2.27463997	10.38420999	8.39565000	#Fe
3.38358998	6.38614999	7.56739000	#Fe
5.29289998	7.63058999	6.45666000	#Fe
3.12865997	11.55221999	6.28067000	#Fe
3.31343998	6.49742999	5.18923000	#Fe
4.28526997	8.83328999	4.25900000	#Fe
2.16686997	10.55789999	3.98133000	#Fe
2.19540998	8.46444999	2.45617000	#Fe
4.23346997	10.22979999	2.13203000	#Fe
3.31278996	12.52556999	1.17406000	#Fe
3.12406998	7.53544999	0.15786000	#Fe
0.93681997	11.52742999	39.84223000	#Fe
5.50113998	6.53738999	38.82386000	#Fe

2.04861997	8.83329999	37.87027000	#Fe
4.38627997	10.59530999	37.54557000	#Fe
4.35914998	8.50400999	36.01009000	#Fe
2.10368997	10.22597999	35.74317000	#Fe
1.13108996	12.56477999	34.80915000	#Fe
5.31896998	7.51195999	33.70507000	#Fe
3.11141997	11.42010999	33.54515000	#Fe
1.20646996	12.66414999	32.42950000	#Fe
4.48733997	8.66137999	31.59895000	#Fe
2.34528997	10.29105999	31.50096000	#Fe
1.09242998	6.37193999	8.70433000	#Ni
0.92682999	2.56966000	6.52887000	#Ni
-1.27551001	3.91530000	6.28064000	#Ni
3.23023000	0.23721000	3.81611000	#Ni
-1.13144002	6.05148999	2.53928000	#Ni
3.18874999	2.51148000	0.26360000	#Ni
1.00133999	3.84727000	39.73381000	#Ni
1.05906000	0.30536000	37.46282000	#Ni
1.04423998	6.11945999	36.17986000	#Ni
0.91660999	2.43797000	33.71994000	#Ni
-1.25626001	3.78621000	33.46851000	#Ni
-1.08781004	12.67613999	31.29821000	#Ni
3.30631000	0.04274000	8.64193000	#Ni
-1.27173003	8.93689999	6.56432000	#Ni
-3.44558003	10.26943999	6.28143000	#Ni
1.03941998	6.59039999	3.82505000	#Ni
-3.31663004	12.40583999	2.52889000	#Ni
1.00165997	8.86238999	0.26928000	#Ni
-1.18464003	10.19917999	39.73338000	#Ni
-1.12848002	6.65875999	37.46694000	#Ni
-1.14276004	12.47185999	36.17615000	#Ni
-1.26964003	8.79108999	33.72155000	#Ni
-3.44257003	10.13969999	33.46669000	#Ni
1.09920998	6.32340999	31.30075000	#Ni
5.46393998	6.36928999	8.69785000	#Ni
5.29939999	2.55885000	6.53193000	#Ni
3.10114999	3.91373000	6.27439000	#Ni
7.60649000	0.23521000	3.81514000	#Ni
3.24403998	6.05181999	2.53746000	#Ni
7.56321999	2.51117000	0.26438000	#Ni
5.37635999	3.84688000	39.73416000	#Ni
5.43346000	0.30530000	37.46283000	#Ni
5.41865998	6.11942999	36.18000000	#Ni
5.29099999	2.43793000	33.71998000	#Ni
3.11815999	3.78625000	33.46840000	#Ni
3.28657996	12.67610999	31.29809000	#Ni
7.65560000	0.02383000	8.70435000	#Ni
3.11644997	8.92056999	6.53360000	#Ni
0.91997997	10.25965999	6.25643000	#Ni
5.41762998	6.59202999	3.82829000	#Ni

1.05723996 12.40449999 2.52744000 #Ni
5.37668997 8.86259999 0.27042000 #Ni
3.19018997 10.19881999 39.73274000 #Ni
3.24614998 6.65877999 37.46681000 #Ni
3.23163996 12.47194999 36.17618000 #Ni
3.10474997 8.79107999 33.72150000 #Ni
0.93177997 10.13973999 33.46664000 #Ni
5.47361998 6.32340999 31.30073000 #Ni

Fe/NiH-FeOH

H 2 O 1 P 24 Fe 48 Ni 24
1.000000000
8.748798 0.000000 0.000000
-2.187200 6.352773 0.000000
0.000000 0.000000 40.000000
H O P Fe Ni
2 1 24 48 24
Cartesian
0.08598998 5.34621999 9.55825000 #H
3.09487999 2.02439000 11.24043000 #H
2.29524999 2.35074000 10.78972000 #O
0.07779999 1.76292000 8.59260000 #P
2.20852999 4.40304000 8.09006000 #P
-0.05080001 2.19006000 4.78523000 #P
2.21765999 4.83825000 4.24079000 #P
2.22364000 1.63875000 2.11753000 #P
-0.07667001 4.29274000 1.59382000 #P
2.11213999 2.16129000 38.38559000 #P
0.04473999 4.82552000 37.89023000 #P
0.03398000 1.63164000 35.68267000 #P
2.14540999 4.34981000 35.30805000 #P
0.15336999 1.74588000 31.60504000 #P
2.37570999 4.55519000 31.42382000 #P
4.47943999 1.71777000 8.51363000 #P
6.57459999 4.52304000 8.24255000 #P
4.29216999 2.14964000 4.72204000 #P
6.60362999 4.84127000 4.27785000 #P
6.60876000 1.63536000 2.11532000 #P
4.29823999 4.28599000 1.58598000 #P
6.48826999 2.16236000 38.38646000 #P
4.41931999 4.82563000 37.88958000 #P
4.40847000 1.63196000 35.68216000 #P
6.51971999 4.34938000 35.30742000 #P
4.52760999 1.74588000 31.60531000 #P
6.75011999 4.55522000 31.42385000 #P
2.28287999 2.28382000 8.96920000 #Fe
-0.00501001 4.03573000 8.47322000 #Fe
1.21363000 0.00768000 7.57081000 #Fe
3.17056000 1.29697000 6.53486000 #Fe

0.98816998	5.25666999	6.21122000	#Fe
1.17425000	0.25074000	5.21282000	#Fe
2.10389999	2.54917000	4.26518000	#Fe
0.03135999	4.29287000	3.93157000	#Fe
0.05572999	2.17878000	2.48019000	#Fe
2.09734999	3.92992000	2.11442000	#Fe
1.16573998	6.23653999	1.17609000	#Fe
0.98529000	1.23461000	0.16362000	#Fe
-1.20393002	5.22830999	39.83442000	#Fe
3.36214000	0.22902000	38.81932000	#Fe
-0.08807001	2.51916000	37.86852000	#Fe
2.25017999	4.27615000	37.54189000	#Fe
2.22319999	2.18163000	36.00313000	#Fe
-0.03388001	3.89875000	35.73856000	#Fe
-1.00970002	6.23411999	34.80702000	#Fe
3.17639000	1.17233000	33.70115000	#Fe
0.96787999	5.07953999	33.54245000	#Fe
-0.93926002	6.32034999	32.42901000	#Fe
2.34200999	2.31568000	31.59655000	#Fe
0.19879999	3.94502000	31.50205000	#Fe
6.66479999	2.33595000	8.54268000	#Fe
4.40358999	3.97457000	8.33218000	#Fe
3.37506998	6.31592999	7.50772000	#Fe
7.55270000	1.30039000	6.48286000	#Fe
5.38500998	5.23819999	6.23182000	#Fe
5.55040000	0.23036000	5.15521000	#Fe
6.50856999	2.55788000	4.24582000	#Fe
4.42728999	4.29738000	3.91515000	#Fe
4.43190999	2.17333000	2.45997000	#Fe
6.47536999	3.93753000	2.11489000	#Fe
5.54433998	6.23200999	1.17840000	#Fe
5.36240000	1.23464000	0.16198000	#Fe
3.16893998	5.22803999	39.83389000	#Fe
7.73750000	0.22900000	38.82019000	#Fe
4.28693999	2.51863000	37.86753000	#Fe
6.62515999	4.27651000	37.54013000	#Fe
6.59754999	2.18115000	36.00357000	#Fe
4.34035999	3.89884000	35.73848000	#Fe
3.36446998	6.23416999	34.80707000	#Fe
7.55078000	1.17219000	33.70120000	#Fe
5.34212999	5.07961999	33.54249000	#Fe
3.43485998	6.32026999	32.42914000	#Fe
6.71644999	2.31560000	31.59666000	#Fe
4.57311999	3.94500000	31.50230000	#Fe
1.15162998	6.21820999	8.72240000	#Ni
1.06884999	2.60912000	6.69486000	#Ni
-1.17832001	3.93841000	6.20187000	#Ni
3.26732000	0.33597000	3.83657000	#Ni
-1.09363002	6.12643999	2.52400000	#Ni
3.23951999	2.55507000	0.26456000	#Ni

1.05129999 3.89652000 39.72539000 #Ni
1.10658000 0.34450000 37.46186000 #Ni
1.09095998 6.14759999 36.17409000 #Ni
0.95855999 2.44974000 33.71599000 #Ni
-1.21435001 3.79595000 33.46794000 #Ni
1.14033998 6.32995999 31.29571000 #Ni
5.43529998 6.30577999 8.65965000 #Ni
5.38636999 2.58603000 6.52961000 #Ni
3.20813999 3.95851000 6.10547000 #Ni
7.66453000 0.34284000 3.84201000 #Ni
3.28423998 6.12287999 2.50823000 #Ni
7.61702999 2.55505000 0.26985000 #Ni
5.42824999 3.89514000 39.72254000 #Ni
5.48130000 0.34456000 37.46318000 #Ni
5.46467998 6.14749999 36.17367000 #Ni
5.33320999 2.44996000 33.71605000 #Ni
3.15986999 3.79575000 33.46824000 #Ni
5.51474998 6.32999999 31.29614000 #Ni

FeH-Fe/Ni-OH

H 2 O 1 P 24 Fe 48 Ni 24
1.000000000
8.748798 0.000000 0.000000
-2.187200 6.352773 0.000000
0.000000 0.000000 40.000000
H O P Fe Ni
2 1 24 48 24
Cartesian
2.24701999 2.16390000 10.08091000 #H
0.91544999 4.79251000 10.79418000 #H
0.24535998 5.20418999 10.21421000 #O
0.20321999 1.83520000 8.62060000 #P
2.29912999 4.57884000 8.18158000 #P
-0.04969001 2.12643000 4.73014000 #P
2.21450999 4.80966000 4.29886000 #P
2.22789000 1.62210000 2.11633000 #P
-0.07505001 4.28120000 1.61579000 #P
2.11155999 2.14462000 38.39466000 #P
0.04235999 4.80811000 37.90365000 #P
0.03197000 1.61612000 35.69284000 #P
2.14348999 4.33462000 35.31998000 #P
0.15090999 1.72784000 31.61592000 #P
2.37360999 4.53695000 31.43373000 #P
4.47754999 1.83384000 8.55144000 #P
6.63585999 4.61590000 8.26362000 #P
4.31176999 2.11185000 4.71341000 #P
6.59923999 4.81633000 4.30179000 #P

6.60476000	1.62224000	2.11391000	#P
4.29817999	4.27718000	1.60800000	#P
6.48679999	2.14495000	38.39298000	#P
4.41692999	4.80861000	37.90142000	#P
4.40618000	1.61628000	35.69251000	#P
6.51779999	4.33407000	35.31923000	#P
4.52524999	1.72781000	31.61599000	#P
6.74797999	4.53697000	31.43406000	#P
2.35569999	2.45503000	8.59444000	#Fe
0.07656999	4.09750000	8.56328000	#Fe
1.25924000	0.07386000	7.52485000	#Fe
3.17120000	1.31566000	6.50147000	#Fe
0.99544998	5.26948999	6.29174000	#Fe
1.17719000	0.21867000	5.19393000	#Fe
2.12858999	2.53262000	4.26850000	#Fe
0.03469999	4.26976000	3.97527000	#Fe
0.05822999	2.16642000	2.46971000	#Fe
2.09855999	3.92180000	2.13495000	#Fe
1.17185998	6.21900999	1.18483000	#Fe
0.98358000	1.21882000	0.16663000	#Fe
-1.20414002	5.21103999	39.84827000	#Fe
3.36068000	0.21219000	38.82832000	#Fe
-0.08817001	2.50430000	37.87926000	#Fe
2.24683999	4.25955000	37.55493000	#Fe
2.22100999	2.16613000	36.01305000	#Fe
-0.03595001	3.88330000	35.74940000	#Fe
-1.01139002	6.21799999	34.81781000	#Fe
3.17359000	1.15507000	33.71191000	#Fe
0.96496999	5.06206999	33.55357000	#Fe
-0.94138002	6.30232999	32.43907000	#Fe
2.33971999	2.29786000	31.60685000	#Fe
0.19637999	3.92684000	31.51304000	#Fe
6.71595999	2.39839000	8.52344000	#Fe
4.48172999	4.08601000	8.37038000	#Fe
5.55683000	0.08891000	7.55914000	#Fe
7.52526000	1.32033000	6.46136000	#Fe
5.36824998	5.26667999	6.25550000	#Fe
5.55190000	0.21158000	5.17742000	#Fe
6.51616999	2.54312000	4.26303000	#Fe
4.41365999	4.27047000	3.96506000	#Fe
4.43423999	2.16334000	2.46774000	#Fe
6.47287999	3.92324000	2.13139000	#Fe
5.54536998	6.21739999	1.18368000	#Fe
5.35890000	1.21886000	0.16508000	#Fe
3.16925998	5.21099999	39.84623000	#Fe
7.73604000	0.21218000	38.82798000	#Fe
4.28563999	2.50387000	37.87902000	#Fe
6.62234999	4.26033000	37.55336000	#Fe
6.59551999	2.16587000	36.01306000	#Fe
4.33821999	3.88322000	35.74914000	#Fe

3.36273998 6.21810999 34.81772000 #Fe
7.54798000 1.15498000 33.71202000 #Fe
5.33919999 5.06208999 33.55346000 #Fe
3.43277998 6.30230999 32.43926000 #Fe
6.71408999 2.29782000 31.60691000 #Fe
4.57078999 3.92674000 31.51311000 #Fe
1.15140998 6.34885299 8.90675000 #Ni
0.96288999 2.65082000 6.59627000 #Ni
-1.19972001 3.97311000 6.26016000 #Ni
3.27848000 0.30361000 3.83374000 #Ni
-1.08604002 6.10517999 2.53151000 #Ni
3.23663999 2.54454000 0.27448000 #Ni
1.04909999 3.88102000 39.74163000 #Ni
1.10542000 0.32754000 37.47168000 #Ni
1.08942998 6.13154999 36.18554000 #Ni
0.95581999 2.43226000 33.72610000 #Ni
-1.21711001 3.77871000 33.47938000 #Ni
1.13837998 6.31155999 31.30762000 #Ni
7.69521000 0.06580000 8.66211000 #Ni
5.36746999 2.63465000 6.53710000 #Ni
3.18050999 3.94224000 6.19242000 #Ni
7.65660000 0.30908000 3.82819000 #Ni
3.28903998 6.10453999 2.53070000 #Ni
7.61141999 2.54534000 0.27383000 #Ni
5.42483999 3.88095000 39.73793000 #Ni
5.47948000 0.32760000 37.47208000 #Ni
5.46345998 6.13148999 36.18454000 #Ni
5.33020999 2.43260000 33.72611000 #Ni
3.15692999 3.77875000 33.47954000 #Ni
5.51271998 6.31170999 31.30787000 #Ni

Fe/NiH-POH

H 2 O 1 P 24 Fe 48 Ni 24
1.000000000
8.748798 0.000000 0.000000
-2.187200 6.352773 0.000000
0.000000 0.000000 40.000000
H O P Fe Ni
2 1 24 48 24
Cartesian
1.05955999 2.25964000 11.21837000 #H
4.24411998 5.77720999 9.14774000 #H
0.34196000 1.70509000 10.85314000 #O
0.26470999 1.81935000 9.18573000 #P
2.22382999 4.63129000 8.24366000 #P
-0.05215001 2.08421000 4.66099000 #P
2.21356999 4.79981000 4.30041000 #P
2.22884000 1.60677000 2.09127000 #P
-0.07877001 4.26984000 1.59671000 #P

4.49756999	1.87238000	8.54276000	#P
6.72453999	4.61672000	8.29955000	#P
4.32637999	2.09423000	4.69717000	#P
6.58902999	4.79832000	4.28335000	#P
6.59817000	1.60898000	2.09030000	#P
4.29585999	4.26933000	1.59811000	#P
2.10928999	2.12955000	38.37450000	#P
0.03892999	4.79252000	37.88343000	#P
0.02762000	1.60063000	35.67295000	#P
2.13843999	4.31824000	35.29916000	#P
0.14525999	1.71561000	31.59726000	#P
2.36927999	4.52459000	31.41231000	#P
6.48236999	2.12876000	38.37318000	#P
4.41306999	4.79270000	37.88312000	#P
4.40191000	1.60057000	35.67314000	#P
6.51278999	4.31794000	35.29883000	#P
4.51963999	1.71561000	31.59744000	#P
6.74353999	4.52458000	31.41223000	#P
2.32332999	2.45897000	8.44301000	#Fe
0.11747999	3.95929000	8.37874000	#Fe
1.21889000	0.16996000	7.58294000	#Fe
3.14885000	1.32284000	6.43285000	#Fe
0.97863998	5.25561999	6.26154000	#Fe
1.16257000	0.19199000	5.18739000	#Fe
2.14258999	2.52597000	4.23661000	#Fe
0.02647999	4.25101000	3.96795000	#Fe
0.05774999	2.15344000	2.43198000	#Fe
2.09701999	3.91413000	2.11616000	#Fe
1.17122998	6.20291999	1.16932000	#Fe
0.98070000	1.20336000	0.14335000	#Fe
6.79355999	2.39276000	8.51155000	#Fe
4.48111999	4.15131000	8.41588000	#Fe
5.59099000	0.08233000	7.61485000	#Fe
7.50484000	1.34152000	6.42672000	#Fe
5.36165998	5.24508999	6.25644000	#Fe
5.54952000	0.19340000	5.20585000	#Fe
6.50742999	2.52656000	4.24733000	#Fe
4.40407999	4.25116000	3.96864000	#Fe
4.42984999	2.15266000	2.44898000	#Fe
6.46945999	3.90981000	2.11424000	#Fe
5.54405998	6.20441999	1.16524000	#Fe
5.35440000	1.20316000	0.14611000	#Fe
-1.20767002	5.19568999	39.82957000	#Fe
3.35705000	0.19577000	38.81084000	#Fe
-0.09074001	2.48939000	37.85927000	#Fe
2.24344999	4.24479000	37.53507000	#Fe
2.21698999	2.14997000	35.99433000	#Fe
-0.04136001	3.86786000	35.72898000	#Fe
-1.01572002	6.20283999	34.79772000	#Fe
3.16951000	1.14189000	33.69181000	#Fe

0.96048999 5.04926999 33.53351000 #Fe
-0.94718002 6.28960999 32.41918000 #Fe
2.33444999 2.28508000 31.58716000 #Fe
0.19218999 3.91441000 31.49169000 #Fe
3.16726998 5.19596999 39.83021000 #Fe
7.73130000 0.19608000 38.80852000 #Fe
4.28271999 2.48931000 37.86012000 #Fe
6.61818999 4.24475000 37.53468000 #Fe
6.59134999 2.14984000 35.99436000 #Fe
4.33291999 3.86779000 35.72947000 #Fe
3.35879998 6.20298999 34.79792000 #Fe
7.54405000 1.14182000 33.69157000 #Fe
5.33490999 5.04918999 33.53343000 #Fe
3.42740998 6.28957999 32.41918000 #Fe
6.70872999 2.28509000 31.58686000 #Fe
4.56655999 3.91431000 31.49184000 #Fe
3.27716000 0.06850000 8.68871000 #Ni
0.95716999 2.60332000 6.49919000 #Ni
-1.22450001 3.96241000 6.24000000 #Ni
3.27322000 0.28594000 3.82247000 #Ni
-1.08650002 6.09075999 2.51940000 #Ni
3.23393999 2.53101000 0.25491000 #Ni
7.78463000 0.09798000 8.62286000 #Ni
5.34206999 2.64657000 6.55549000 #Ni
3.15622999 3.95512000 6.23537000 #Ni
7.64299000 0.28527000 3.80714000 #Ni
3.28690998 6.09125999 2.52818000 #Ni
7.60581999 2.53231000 0.24915000 #Ni
1.04564999 3.86529000 39.72223000 #Ni
1.10115000 0.31141000 37.45266000 #Ni
1.08462998 6.11579999 36.16497000 #Ni
0.95238999 2.41951000 33.70779000 #Ni
-1.22117001 3.76658000 33.45747000 #Ni
1.13248998 6.29907999 31.28553000 #Ni
5.41925999 3.86689000 39.72148000 #Ni
5.47475000 0.31136000 37.45274000 #Ni
5.45931998 6.11557999 36.16479000 #Ni
5.32686999 2.41939000 33.70782000 #Ni
3.15317999 3.76685000 33.45783000 #Ni
5.50688998 6.29909999 31.28526000 #Ni

Fe/NiH-POH_2

H 2 O 1 P 24 Fe 48 Ni 24
1.000000000
8.748798 0.000000 0.000000
-2.187200 6.352773 0.000000
0.000000 0.000000 40.000000
H O P Fe Ni
2 1 24 48 24

Cartesian

-0.03316002	5.55894999	9.33815000 #H
0.02831999	2.37050000	11.28451000 #H
0.43491000	1.63080000	10.79192000 #O
0.21049999	1.81649000	9.13135000 #P
2.32600999	4.58394000	8.26053000 #P
-0.03283001	2.07445000	4.68250000 #P
2.21987999	4.77345000	4.28066000 #P
2.23485000	1.58666000	2.09273000 #P
-0.06904001	4.24978000	1.59508000 #P
2.11579999	2.11359000	38.37346000 #P
0.04666999	4.77698000	37.88230000 #P
0.03320000	1.58844000	35.67046000 #P
2.14397999	4.30585000	35.29707000 #P
0.14889999	1.70912000	31.59599000 #P
2.37232999	4.51802000	31.41075000 #P
4.57198999	1.80830000	8.54768000 #P
6.66831999	4.57885000	8.24616000 #P
4.32573999	2.06919000	4.68578000 #P
6.60948999	4.78000000	4.28725000 #P
6.61085000	1.58697000	2.08955000 #P
4.30419999	4.24501000	1.59285000 #P
6.48987999	2.11335000	38.37290000 #P
4.42082999	4.77706000	37.88161000 #P
4.40750000	1.58844000	35.67061000 #P
6.51843999	4.30556000	35.29680000 #P
4.52333999	1.70918000	31.59619000 #P
6.74669999	4.51814000	31.41081000 #P
2.34789999	2.39337000	8.48609000 #Fe
0.12896999	4.03122000	8.43478000 #Fe
1.24924000	0.12707000	7.68357000 #Fe
3.16029000	1.28502000	6.45346000 #Fe
0.99607998	5.21596999	6.26398000 #Fe
1.17717000	0.17092000	5.23347000 #Fe
2.14509999	2.49897000	4.24378000 #Fe
0.03977999	4.22951000	3.95899000 #Fe
0.06602999	2.13165000	2.44008000 #Fe
2.10364999	3.88754000	2.11502000 #Fe
1.17640998	6.18395999	1.16570000 #Fe
0.98967000	1.18295000	0.14430000 #Fe
-1.19786002	5.17646999	39.82813000 #Fe
3.36438000	0.17967000	38.80916000 #Fe
-0.08467001	2.47340000	37.85732000 #Fe
2.25083999	4.22982000	37.53293000 #Fe
2.22200999	2.13666000	35.99238000 #Fe
-0.03589001	3.85585000	35.72738000 #Fe
-1.00960002	6.19156999	34.79620000 #Fe
3.17465000	1.13293000	33.69006000 #Fe
0.96562999	5.04075999	33.53168000 #Fe
-0.94339002	6.28241999	32.41748000 #Fe

2.33804999 2.27825000 31.58500000 #Fe
0.19558999 3.90784000 31.48882000 #Fe
6.81224999 2.38734000 8.47575000 #Fe
4.50567999 4.04972000 8.36130000 #Fe
5.60781000 0.04496000 7.51896000 #Fe
7.52182000 1.29893000 6.41082000 #Fe
5.37764998 5.21306999 6.23959000 #Fe
5.55365000 0.16728000 5.15751000 #Fe
6.52511999 2.50720000 4.23639000 #Fe
4.41595999 4.23166000 3.95935000 #Fe
4.43898999 2.12952000 2.44355000 #Fe
6.47978999 3.89152000 2.10996000 #Fe
5.55491998 6.18237999 1.16533000 #Fe
5.36419000 1.18415000 0.14476000 #Fe
3.17455998 5.17717999 39.82769000 #Fe
7.73968000 0.17912000 38.80790000 #Fe
4.28947999 2.47332000 37.85708000 #Fe
6.62565999 4.22976000 37.53236000 #Fe
6.59627999 2.13638000 35.99239000 #Fe
4.33829999 3.85576000 35.72757000 #Fe
3.36473998 6.19161999 34.79644000 #Fe
7.54914000 1.13287000 33.68999000 #Fe
5.34005999 5.04073999 33.53174000 #Fe
3.43099998 6.28230999 32.41763000 #Fe
6.71245999 2.27838000 31.58499000 #Fe
4.57002999 3.90787000 31.48905000 #Fe
3.38946000 0.00438000 8.73996000 #Ni
0.97111999 2.58131000 6.54923000 #Ni
-1.19453001 3.91867000 6.23754000 #Ni
3.27591000 0.26293000 3.81839000 #Ni
-1.07767002 6.07076999 2.52835000 #Ni
3.24208999 2.51147000 0.25244000 #Ni
1.05471999 3.84630000 39.71988000 #Ni
1.10868000 0.29634000 37.45114000 #Ni
1.09181998 6.10327999 36.16457000 #Ni
0.95789999 2.41075000 33.70644000 #Ni
-1.21561001 3.75793000 33.45500000 #Ni
1.13612998 6.29264999 31.28426000 #Ni
7.72141000 0.06374000 8.58352000 #Ni
5.36078999 2.59057000 6.51965000 #Ni
3.16926999 3.92552000 6.21398000 #Ni
7.66231000 0.26667000 3.80833000 #Ni
3.29515998 6.06837999 2.51065000 #Ni
7.61689999 2.51184000 0.25025000 #Ni
5.42910999 3.84739000 39.71824000 #Ni
5.48266000 0.29614000 37.45219000 #Ni
5.46616998 6.10305999 36.16408000 #Ni
5.33241999 2.41063000 33.70648000 #Ni
3.15871999 3.75792000 33.45521000 #Ni
5.51038998 6.29269999 31.28428000 #Ni

PH-Ni/FeOH

```
H 2   O 1   P 24   Fe 48   Ni 24
  1.000000000
    8.748798     0.000000     0.000000
    -2.187200     6.352773     0.000000
    0.000000     0.000000     40.000000
H   O   P   Fe   Ni
2   1   24   48   24
Cartesian
0.42198000     1.57548000     10.48850000  #H
5.85116000     0.48752000     10.36752000  #H
6.73266000     0.88286000     10.21762000  #O
0.34361999     1.72991000     9.05910000  #P
2.28132999     4.59882000     8.30269000  #P
-0.05401001     2.05728000     4.68227000  #P
2.22970999     4.77263000     4.28957000  #P
2.23911000     1.58606000     2.09357000  #P
-0.06935001     4.24686000     1.59618000  #P
4.46975999     1.78349000     8.48431000  #P
6.68055999     4.50574000     8.27698000  #P
4.33982999     2.08909000     4.71543000  #P
6.59688999     4.77405000     4.28927000  #P
6.60913000     1.58866000     2.09740000  #P
4.30727999     4.24502000     1.59841000  #P
2.11422999     2.11704000     38.37731000  #P
0.04421999     4.78016000     37.88449000  #P
0.02926000     1.59101000     35.67359000  #P
2.14005999     4.30812000     35.29933000  #P
0.14547999     1.71026000     31.59741000  #P
2.36902999     4.51923000     31.41233000  #P
6.48719999     2.11600000     38.37638000  #P
4.41860999     4.77980000     37.88482000  #P
4.40360000     1.59097000     35.67378000  #P
6.51439999     4.30793000     35.29935000  #P
4.51994999     1.71027000     31.59756000  #P
6.74334999     4.51934000     31.41206000  #P
2.35679999     2.42835000     8.40971000  #Fe
0.11716999     3.91379000     8.36201000  #Fe
1.16535000     0.05685000     7.57182000  #Fe
3.16293000     1.26851000     6.41899000  #Fe
0.99835998     5.20332999     6.26107000  #Fe
1.17953000     0.16639000     5.18687000  #Fe
2.15097999     2.49302000     4.22873000  #Fe
0.04175999     4.22399000     3.96401000  #Fe
0.06574999     2.12778000     2.44074000  #Fe
2.10467999     3.89184000     2.11776000  #Fe
1.17825998     6.18332999     1.16881000  #Fe
0.98990000     1.18580000     0.14767000  #Fe
```

6.72177999	2.25554000	8.76512000	#Fe
4.47900999	4.03273000	8.39421000	#Fe
5.60010000	0.01853000	7.50014000	#Fe
7.56170000	1.30763000	6.53058000	#Fe
5.39374998	5.22242999	6.29667000	#Fe
5.56664000	0.17456000	5.16888000	#Fe
6.52244999	2.49820000	4.25827000	#Fe
4.41893999	4.23198000	3.95492000	#Fe
4.44111999	2.12846000	2.45651000	#Fe
6.48018999	3.88654000	2.11833000	#Fe
5.55433998	6.18475999	1.16686000	#Fe
5.36323000	1.18547000	0.14986000	#Fe
-1.20001002	5.17882999	39.83132000	#Fe
3.36300000	0.18246000	38.81227000	#Fe
-0.08699001	2.47655000	37.86012000	#Fe
2.24895999	4.23296000	37.53505000	#Fe
2.21811999	2.13918000	35.99515000	#Fe
-0.03986001	3.85793000	35.72962000	#Fe
-1.01403002	6.19409999	34.79844000	#Fe
3.17024000	1.13498000	33.69235000	#Fe
0.96128999	5.04235999	33.53387000	#Fe
-0.94700002	6.28373999	32.41937000	#Fe
2.33448999	2.27955000	31.58776000	#Fe
0.19194999	3.90902000	31.49113000	#Fe
3.17492998	5.17859999	39.83112000	#Fe
7.73651000	0.18235000	38.81112000	#Fe
4.28679999	2.47618000	37.86062000	#Fe
6.62314999	4.23290000	37.53513000	#Fe
6.59247999	2.13931000	35.99567000	#Fe
4.33450999	3.85805000	35.73047000	#Fe
3.36064998	6.19423999	34.79874000	#Fe
7.54469000	1.13511000	33.69232000	#Fe
5.33573999	5.04237999	33.53396000	#Fe
3.42758998	6.28371999	32.41939000	#Fe
6.70878999	2.27968000	31.58757000	#Fe
4.56643999	3.90898000	31.49134000	#Fe
1.08821998	6.34373999	8.66142000	#Ni
1.00163999	2.57074000	6.48487000	#Ni
-1.19347001	3.92172000	6.21136000	#Ni
3.28971000	0.26319000	3.82021000	#Ni
-1.07573002	6.06848999	2.52120000	#Ni
3.24353999	2.51369000	0.25873000	#Ni
5.52444998	6.31513999	8.78932000	#Ni
5.40899999	2.58210000	6.57694000	#Ni
3.18580999	3.91794000	6.22209000	#Ni
7.66098000	0.26345000	3.81077000	#Ni
3.29674998	6.07038999	2.51515000	#Ni
7.61590999	2.51398000	0.25342000	#Ni
1.05410999	3.84876000	39.72137000	#Ni
1.10570000	0.29957000	37.45459000	#Ni

1.08770998 6.10564999 36.16640000 #Ni
0.95318999 2.41279000 33.70854000 #Ni
-1.22017001 3.76041000 33.45720000 #Ni
1.13262998 6.29382999 31.28626000 #Ni
5.42875999 3.84951000 39.72178000 #Ni
5.47987000 0.29908000 37.45442000 #Ni
5.46252998 6.10563999 36.16639000 #Ni
5.32773999 2.41279000 33.70869000 #Ni
3.15436999 3.76034000 33.45753000 #Ni
5.50694998 6.29386999 31.28595000 #Ni

PH-Ni/FeOH_2

H 2 O 1 P 24 Fe 48 Ni 24
1.000000000
8.748798 0.000000 0.000000
-2.187200 6.352773 0.000000
0.000000 0.000000 40.000000
H O P Fe Ni
2 1 24 48 24
Cartesian
0.07047999 1.70901000 10.66061000 #H
1.86112000 0.67219000 10.71809000 #H
2.64772000 1.06996000 10.29924000 #O
2.10704999 2.16888000 38.36601000 #P
0.03700999 4.83248000 37.87529000 #P
0.02323000 1.65097000 35.66230000 #P
2.13464999 4.36705000 35.28998000 #P
0.14278999 1.77440000 31.59050000 #P
2.36647999 4.58275000 31.40493000 #P
0.03818999 1.82609000 9.21962000 #P
2.25120999 4.54608000 8.25564000 #P
-0.03394001 2.12754000 4.69989000 #P
2.20330999 4.80954000 4.28045000 #P
2.22336000 1.62793000 2.08601000 #P
-0.07693001 4.28773000 1.58796000 #P
6.48212999 2.16980000 38.36564000 #P
4.41104999 4.83306000 37.87393000 #P
4.39741000 1.65106000 35.66241000 #P
6.50914999 4.36705000 35.28936000 #P
4.51724999 1.77435000 31.59058000 #P
6.74078999 4.58281000 31.40506000 #P
4.53728999 1.84979000 8.35688000 #P
6.68403999 4.62379000 8.31104000 #P
4.30735999 2.08811000 4.66308000 #P
6.60298999 4.81786000 4.27377000 #P
6.60498000 1.62501000 2.07900000 #P
4.29351999 4.28366000 1.58138000 #P
-1.20644002 5.22511999 39.82113000 #Fe
3.35551000 0.23234000 38.80246000 #Fe

-0.09394001	2.52835000	37.84824000	#Fe
2.24102999	4.28849000	37.52517000	#Fe
2.21193999	2.19746000	35.98745000	#Fe
-0.04487001	3.91784000	35.72191000	#Fe
-1.01810002	6.25452999	34.79018000	#Fe
3.16688000	1.19664000	33.68360000	#Fe
0.95822999	5.10538999	33.52512000	#Fe
-0.94898002	6.34743299	32.41113000	#Fe
2.33154999	2.34333000	31.57873000	#Fe
0.18951999	3.97343000	31.48262000	#Fe
2.25011999	2.30565000	8.76401000	#Fe
0.10075999	3.96148000	8.41931000	#Fe
1.23663000	0.15396000	7.60518000	#Fe
3.15617000	1.33240000	6.47853000	#Fe
0.99266998	5.27102999	6.29722000	#Fe
1.16823000	0.21662000	5.21302000	#Fe
2.13090999	2.53344000	4.24373000	#Fe
0.02894999	4.26904000	3.95282000	#Fe
0.05659999	2.17052000	2.44209000	#Fe
2.09434999	3.92691000	2.10922000	#Fe
1.16877998	6.22672999	1.15808000	#Fe
0.98050000	1.23181000	0.13655000	#Fe
3.16556998	5.22734999	39.82041000	#Fe
7.73138000	0.23246000	38.80074000	#Fe
4.27945999	2.52858000	37.84796000	#Fe
6.61660999	4.28833000	37.52388000	#Fe
6.58628999	2.19717000	35.98659000	#Fe
4.32938999	3.91757000	35.72145000	#Fe
3.35597998	6.25448999	34.79033000	#Fe
7.54135000	1.19646000	33.68344000	#Fe
5.33272999	5.10518999	33.52487000	#Fe
3.42551998	6.34728299	32.41145000	#Fe
6.70592999	2.34340000	31.57880000	#Fe
4.56391999	3.97329000	31.48268000	#Fe
6.73446999	2.41732000	8.43272000	#Fe
4.46901999	4.08937000	8.33316000	#Fe
5.54750000	0.02988000	7.49778000	#Fe
7.50523000	1.33866000	6.39519000	#Fe
5.36048998	5.25171999	6.21858000	#Fe
5.54732000	0.20637000	5.14109000	#Fe
6.52946999	2.54230000	4.21298000	#Fe
4.40978999	4.26096000	3.95450000	#Fe
4.43021999	2.16424000	2.42962000	#Fe
6.46832999	3.93274000	2.10013000	#Fe
5.54577998	6.22397999	1.15629000	#Fe
5.35646000	1.23339000	0.13559000	#Fe
1.04660999	3.89639000	39.71071000	#Ni
1.10055000	0.35253000	37.44457000	#Ni
1.08360998	6.16503999	36.15785000	#Ni
0.95087999	2.47535000	33.70068000	#Ni

-1.22246001	3.82215000	33.44843000	#Ni
3.31828000	0.00500000	31.27718000	#Ni
3.36814000	0.00648000	8.84702000	#Ni
1.00760999	2.60444000	6.58265000	#Ni
-1.19962001	3.97431000	6.24460000	#Ni
3.26429000	0.29569000	3.80836000	#Ni
-1.08731002	6.10998999	2.51261000	#Ni
3.23322999	2.55857000	0.24500000	#Ni
5.42048999	3.89784000	39.70705000	#Ni
5.47445000	0.35306000	37.44583000	#Ni
5.45765998	6.16491999	36.15734000	#Ni
5.32515999	2.47523000	33.70044000	#Ni
3.15177999	3.82249000	33.44847000	#Ni
7.69252000	0.00500000	31.27690000	#Ni
7.68828000	0.07242000	8.53938000	#Ni
5.36269999	2.68865000	6.43707000	#Ni
3.15132999	3.97474000	6.18826000	#Ni
7.66035000	0.30573000	3.80105000	#Ni
3.28978998	6.10507999	2.50516000	#Ni
7.61070999	2.55867000	0.24520000	#Ni
